# Glycyrrhizic Acid and Its Hydrolyzed Metabolite 18β-Glycyrrhetinic Acid as Specific Ligands for Targeting Nanosystems in the Treatment of Liver Cancer

**DOI:** 10.3390/pharmaceutics13111792

**Published:** 2021-10-26

**Authors:** Luciano A. Stecanella, Antonio P. R. Bitencourt, Gustavo Richter Vaz, Eride Quarta, José O. C. Silva Júnior, Alessandra Rossi

**Affiliations:** 1 Laboratory R&D Pharmaceutical and Cosmetic, Federal University of Pará, Street Augusto Correa 01, Belém 66075-110, Brazil; lastecanella@ufpa.br (L.A.S.); antoniopaulo.ribeirobitencourt@unipr.it (A.P.R.B.); carrera@ufpa.br (J.O.C.S.J.); 2 Department of Food and Drug, University of Parma, Parco Area delle Scienze 27/A, 43124 Parma, Italy; gustavo.vaz@unipr.it (G.R.V.); eride.quarta@studenti.unipr.it (E.Q.); 3 Laboratory of Nanotechnology Applied to Health, Postgraduate Program in Health Sciences, Federal University of Rio Grande, Av. Italia, Km 8, Rio Grande 96210-900, Brazil; 4 Department of Food and Drug, Plumestars srl, c/o Parco Area delle Scienze 27/A, 43124 Parma, Italy; 5 Interdepartmental Center for Innovation in Health Products, Biopharmanet-TEC, University of Parma, Parco Area delle Scienze 27/A, 43124 Parma, Italy

**Keywords:** glycyrrhizic acid, glycyrrhetinic acid, liver cancer, nanosystems, drug targeting

## Abstract

Glycyrrhizic acid and its hydrolyzed metabolite 18β-glycyrrhetinic acid, obtained from the plant *Glycyrrhiza glabra*, have numerous pharmacological activities, such as anti-inflammatory, anti-ulcerative, antiallergic, immunomodulatory, antiviral, antitumor, hepatoprotective, and antioxidant effects, and others. In addition to the pharmacological activities, in the 1980s, an interaction and uptake of these molecules by the liver was verified, which was later confirmed by other studies through the discovery of specific receptors in the hepatocytes. The presence of these specific receptors in the liver led to vectorization and delivery of drugs, by the introduction of glycyrrhizic acid or glycyrrhetinic acid on the surface of nanosystems, for the treatment of liver diseases. This review describes experimental evidence of vectorization by conjugating glycyrrhizic acid or glycyrrhetinic acid to nanosystems and delivery of antitumor drugs for the treatment of liver cancer and also describes the techniques used to perform this conjugation. We have shown that due to the existence of specific receptors for these molecules, in addition to the targeting of nanosystems to hepatocytes, nanosystems having glycyrrhizic acid or glycyrrhetinic acid on their surface had the same therapeutic effect in a significantly lower dose compared to the free drug and unconjugated nanosystems, with consequent reduction of side effects and toxicity.

## 1. Introduction

### 1.1. Liver Cancer

Among cancer diseases, in 2018, liver cancer was considered the second highest cause of male mortality with 841,080 cases diagnosed and 781,631 deaths in the same year, being the sixth highest incidence rate by age in the world [1]. Approximately 70–90% of the liver cancer diagnosed in the world is hepatocellular carcinoma (HCC) [2]. Another type of liver cancer, but with a lower incidence, is cholangiocarcinoma, which is also known as bile duct cancer; it is most commonly diagnosed in Thailand and other parts of Asia due to the presence of liver flukes in the consumed raw fish dishes [3].

The major cause of hepatocellular carcinoma is chronic hepatitis B and C, but other possible causes are cirrhosis linked to excessive alcohol consumption, aflatoxin, smoking, type 2 diabetes, and obesity-induced hepatic steatosis [2,4,5,6]. Recent studies predict a rise in the incidence of liver cancer by 2030 due to increased alcoholism and obesity [7].

So far, the treatment of liver cancers may include hepatic resection, liver transplantation, transarterial chemoembolization, ablation, first-line and second-line drug therapies, depending on the HCC stage [8].

Specific drug delivery systems that can target the drugs into the tumor site have demonstrated great potential in cancer treatment [9]. With regard to that, there has been a great interest from researchers in using glycyrrhizic acid and its derivative as drug delivery carriers for the treatment of HCC, also on the basis of their hepatoprotective activity exploited for over thirty years for the treatment of liver disease in Asia [10,11].

This review is focused on an in-depth analysis of the research strategies to obtain glycyrrhizic acid or glycyrrhetinic acid-conjugated drug delivery systems that are able to target the drug in the HCC and reduce drug side effects and toxicity.

### 1.2. Glycyrrhizic Acid and 18β-Glycyrrhetinic Acid

**Figure 1:**
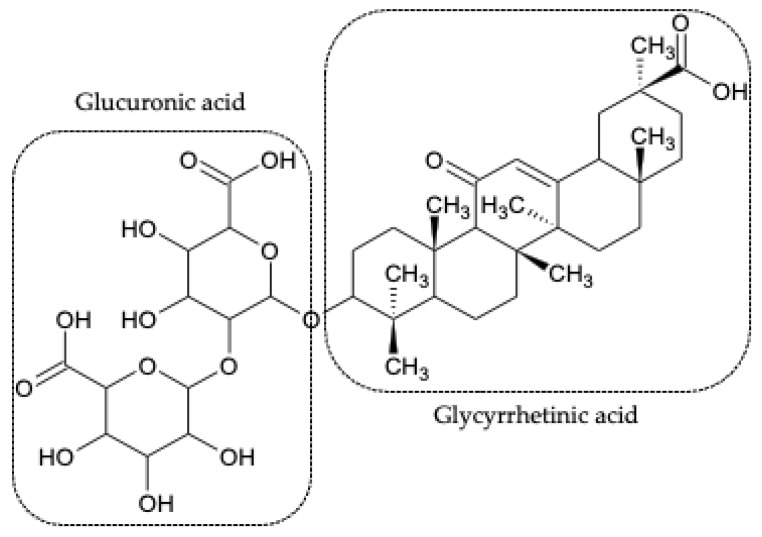
Chemical structure of the triterpenic saponin glycyrrhizic acid (GL) showing the two molecules of glucuronic acid and the molecule of glycyrrhetinic acid (GA).

Glycyrrhizic acid, also known as glycyrrhizin (GL) (Figure 1), is a triterpenic saponin obtained from *Glycyrrhiza glabra* [12] and is composed of two molecules of glucuronic acid and one molecule of glycyrrhetinic acid (GA). GA is obtained from hydrolysis of GL, both of them being major compounds of the Glycyrrhiza glabra L. root extract [13]. It has also been found that GA is the product of the biotransformation of GL by human intestinal flora [14]. In agreement with published studies, even at high concentrations, GL has pharmacological tolerance and absence of cytotoxicity in rats and humans [15,16]. Even GA, when given intravenously at a dose of 240 mg three times a week for 4 weeks, is well tolerated by humans, and it has no adverse effects [16,17].

GL and GA have numerous pharmacological activities, such as anti-inflammatory [18,19,20], anti-ulcerative, antiallergic, immunomodulatory [21,22], antiviral [23,24,25], antileishmanial [26], antitumor [27,28,29,30,31], hepatoprotective [13,32,33,34,35], antioxidant [36,37,38], cardioprotective [38,39,40], and neuroprotective effects [41,42,43]. Moreover, they protect the brain tissue in global ischemia, brain lesions induced by intracerebral hemorrhage, and focal ischemia [12]. In addition to the described activities, a study suggested that GL may be a potential therapeutic agent in the treatment of lymphoid malignancies associated with the Epstein–Barr virus [44]. On the other side, GA studies have demonstrated that GA alone shows therapeutic potential in the treatment of multiple sclerosis [45].

Apart from their therapeutic activities, other uses have been discovered for GL and GA due to the amphiphilic characteristic of the molecules: (i) ability to self-associate in aqueous and non-aqueous media forming micelles and other structures; (ii) increasing the solubility of lipophilic drugs through the formation of complexes; (iii) modification of the lipid bilayer making the cell membrane more flexible and permeable. Thus, due to their ability to interact with the cell membrane, the entry of drugs into the cells can be facilitated, proving to be an efficient drug delivery system [46].

#### Pharmacological Mechanism

The ammonium salt of glycyrrhizic acid stands out for its anti-inflammatory activity [47,48]. The anti-inflammatory mechanism involves (1) cytokines and chemokines such as TNF-α (tumor necrosis factor-alpha), IL-1β (interleukin-1β), IL-18 (interleukin-18), IL-17 (interleukin-17), IL-12 (interleukin-12), IL- 10 (interleukin-10), IL-8 (interleukin-8), IL-6 (interleukin-6), IL-5 (interleukin-5), IL-4 (interleukin-4), IFN-γ (interferon-γ), and eotaxin-1 (eosinophil-specific chemoattractant); (2) ICAM-1 (intercellular cell adhesion molecule) and P-selectin (type-1 transmembrane protein); (3) some enzymes such as iNOS (inducible nitric oxide synthase); and (4) transcription factors, such as NF-kB (nuclear factor kappa-light-chain-enhancer of activated B cells), STAT-3 (signaling transducer and activator of transcription 3), and STAT6 (signaling transducer and activator of transcription 6) [49].

**Figure 2:**
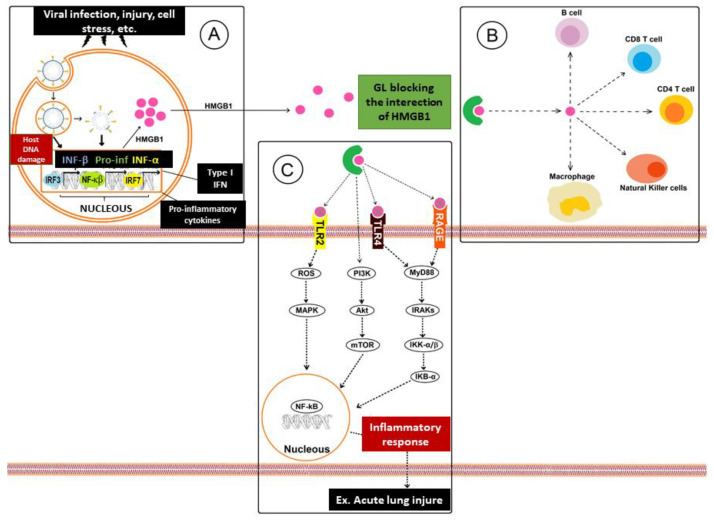
Mechanism of inhibitory action of glycyrrhizic acid in HMGB1 protein-mediated infectious diseases: (**A**) cells with active replicating virus become apoptotic and die, releasing type I IFN, HMGB1, and pro-inflammatory cytokines in the extracellular environment; (**B**) HMGB1, actively released after a viral infection, major stress, injury, etc., also acts as a cytokine that plays a modulating role in innate and adaptive immune responses, stimulating NK immune cells, macrophages, and B and T cells; (**C**) HMGB1 present in the extracellular environment binds to specific receptors on the surface of other cells, such as RAGE, TLR2, and TLR4, inducing the production of inflammatory cytokines, chemokines, adhesion molecules, and ROS, thus activating the signal of protein kinase pathways by NF-kB and MAPK (mitogen-activated protein kinase) to mediate inflammatory molecules.

TNF-α, IL-1β, and HMGB1 (high mobility group box 1) are pro-inflammatory factors present in the process of many infectious diseases. HMGB1, released in infectious processes, generates severe inflammatory processes due to its connection with several receptors such as TLR2 (toll-like receptor 2), TLR4 (toll-like receptor 4), and RAGE (receptor of advanced glycation end products); this leads to activation of the kinase pathway associated with the IL-1 receptor via the MyD88 (myeloid differentiation factor 88) [19,50,51]. Activation of IKK-a/IKK-b (inhibitor of nuclear factor kappa-B kinase subunit alpha/inhibitor of nuclear factor kappa-B kinase subunit beta) leads to phosphorylation and degradation of IkB-α (nuclear factor of kappa light polypeptide gene enhancer in B-cells inhibitor, alpha), which in turn activates even more NF-kB and the release of pro-inflammatory cytokines, including TNF-α, IL-1β, and IL-6, to induce inflammation via the PI3K/AKT/mTOR (phosphoinositide 3-kinases/protein kinase B/mechanistic target of rapamycin) pathway [19]. Consequently, GL could be useful for the treatment of acute lung injury (ALI) and also for the syndromes from acute respiratory distress [49] and immunoglobulin E-mediated allergic reaction [22]. In addition to these inflammatory mechanisms, HMGB1 stimulates the effector function of immune cells, such as lymphoid and myeloid cells, including natural killer cells (NK), macrophages, and B and T cells, also showing the immunomodulatory action of GL [52,53]. A study showed that GL inhibits HMGB1 and suppresses the secretion of TNF-α and IL-1β, which are inflammatory cytokines released in ALI models in mice [43,51,54,55]. Moreover, GL effectively attenuated thyroiditis by inhibition of TLR2-HMGB1 signaling [56] and reduced ferroptosis level during acute liver failure through the inhibition of oxidative stress pathways due to GL capacity on reducing HMGB1 and ROS (reactive oxygen species) [57]. The immunomodulatory action of GL is schematically represented in Figure 2.

**Figure 3:**
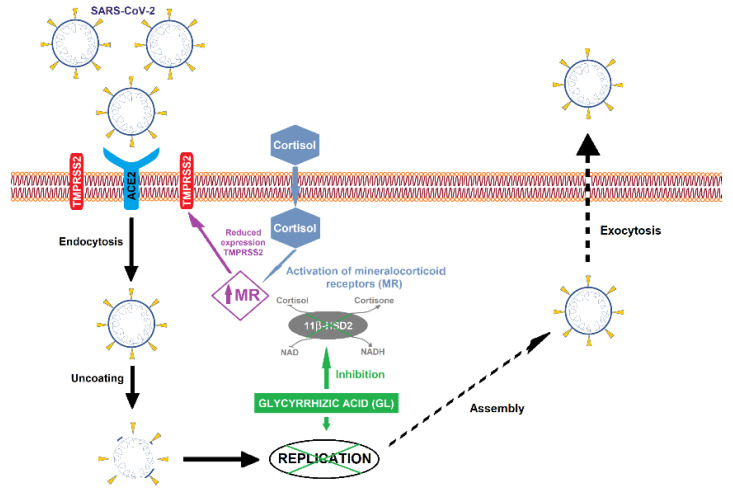
Schematic representation of glycyrrhizic acid effect on SARS-CoV-2 virus replication. The penetration of the SARS-CoV-2 virus into the cells, where it finds the ideal conditions for its replication, is mediated by ACE2 receptor with TMPRSS2 co-factor. Since ACE2 expression is regulated by MR (mineralocorticoid receptors), GL (green box) inside the cell inhibits 11β-HSD2 enzyme, allowing cortisol to activate MR. Then, the activation of MR leads to reduction of TMPRSS2 expression, causing an ACE2 downregulation. The 11β-HSD2 inhibition restricts the virus access to the cell, and consequently, the virus replication is inhibited.

The potential clinical use of GL has also been observed in the treatment of acute viral hepatitis [58]. Moreover, assessing its potential use as an antiviral, GL has the potential to be used for the treatment of SARS-CoV-2 (severe acute respiratory syndrome coronavirus 2) virus [59,60]. With regard to this, GL can act in two different ways. As schematically described in Figure 3, the first way is related to the reduction in the expression of the co-factor known as TMPRSS2 (transmembrane serine protease type 2) through the direct inhibition of 11β-HSD2 (11β-hydroxysteroid dehydrogenase type 2 enzyme) and consequent decrease in ACE2 (angiotensin-converting enzyme 2), which is linked to the interaction of several organs (lung, heart, liver, and kidneys) with the SARS-CoV-2 [61,62,63]. The second pathway is related to its antiviral activity [23]. GL induces nitrous oxide synthase in cells, causing inhibition of virus replication. In addition, GL affects cellular signaling pathways, such as protein kinase C, casein kinase II, and transcription factors, such as activating protein 1 and NF-kB.

### 1.3. Vectorization of Nanosystems

The direct cellular delivery of most compounds is limited by the lipophilic characteristics of the cell membrane. Simple diffusion is restricted only to molecules smaller than 1 KDa (small molecules and ions), requiring administration at the intracellular level to carry out its therapeutic action [64]. For this reason, a great evolution has occurred in the development of drug delivery systems with the use of nanosystems with biocompatible materials, thus improving pharmacokinetics and reducing cytotoxicity [65].

Nanotechnology is being studied for use in all therapeutic classes, but major emphasis is given to anticancer drugs because of their extensive side effects caused by the non-selectivity of the drugs to the cancer cells. For this reason, nanocarriers have been designed to reach only the cancer cells, in particular the constituents of the structure as proteins, peptides, nucleic acids, polysaccharides, and lipids, alone or in combination. The nanosystem’s selective activity allows the intracellular release of the drug by enhancing drug efficacy, especially in the case of multidrug resistance [64]. This transport or selective delivery is called vectorization [66].

An example of vectorization is the use of cellular penetration peptides, which are inserted into the surface of the nanoparticles, through which cell membrane translocation occurs, and consequently, the penetration efficiency increases. Thus, the internalization of the nanoparticles favors the impact reduction on patients’ health [64]. This internalization was observed with liposomes decorated with glycyrrhizic acid due to the presence of GL receptors on the surface of rat hepatocytes [67]. The increasing evolution of the molecular biology knowledge and the consequent discovery of new targets and promising target ligands provides new information for the development of new nanosystems decorated for vectorization [68,69,70].

## 2. Glycyrrhizic Acid and Glycyrrhetinic Acid Receptors

The affinity of GA and GL for the receptors in the liver was demonstrated by several researchers. In a study conducted in 1986, Ichikawa et al. found that about 80% of the GL dose intravenously administered to rats was excreted in bile, and this prompted to carry out the development of new products aimed at the delivering of the drug to the liver, based on the hypothesis that GL could have specific receptors in the liver [71]. Tsuji et al. introduced GL on the liposome surface in order to obtain a new colloidal system that allowed the drug delivery to the liver [72]. It was verified an accumulation in the liver of 42% of intravenously injected dose after four hours of its application, thus suggesting the presence of specific sites for GL in the liver. Subsequently, in 1994, the same research group published an article in which the presence of specific receptors for GL in the liver was confirmed [67]. In this work, primary cultures of rat hepatocytes were placed in contact with a 50 mM solution of GL, causing saturation of the receptors and reducing the uptake of GL-decorated liposomes by 80%. This effect was not observed with the undecorated liposome, thus confirming that the receptors were GL binding site-specific. In order to understand which part of the GL molecule bound to the receptor, a test was performed with the cells pre-incubated with a 20 mM glucuronic acid solution. As the GL-decorated liposome uptake was not reduced, the authors concluded that the portion bound to the receptor was GA.

In summary, these works have opened the door for the development of new products for the treatment of liver diseases, among which many are fatal, and the treatments available are with drugs that are poorly absorbed in the hepatocytes and consequently have low efficiency [30].

## 3. Functionalized Nanoparticles with Glycyrrhizic Acid (GL) or Glycyrrhetinic Acid (GA)

In recent years there has been an exponential increase in studies with nanoparticle vectors, mainly focused on the treatment of liver cancer. GL and GA have been highlighted by their specificity for healthy or cancerous liver cells, referring to the ligand group widely used in the vectorization of micelles containing anticancer drugs.

With the discovery of specific hepatic cell receptors for GL and its metabolite (GA) in the 1980s and its combination with nanosystems, a new opportunity was opened up for liver disease therapies, including liver cancer. Therefore, through the union of the nanosystems with GL or GA, it was possible to obtain site-specific delivery systems. New studies with GL- or GA-functionalized nanosystems focusing on liver cancer have been performed in the last 10 years. The nanosystems studied have been liposomes, nanocapsules, solid nanoparticles, and other less used macromolecules, such as dendrimers. Examples of GL- and GA-functionalized nanosystems for liver drug targeting are summarized in Tables S1 and S2 in the Supplementary Materials.

Liposomes or solid particles nanosystems containing GA on the surface have been developed by various methods, which generate micelles with sizes between 110 and 200 nm [73,74,75,76,77,78]. Since for cancer treatment, the ideal size requirement for particles is between 70 and 200 nm, the appropriate size and good dispersion would be favorable to evade the body reticuloendothelial system and accumulate in tumor tissues by EPR (enhanced permeability and retention) effect [79]. In addition to the determination of average particle size performed by dynamic light scattering (DLS) and transmittance electronic microscopy (TEM), other techniques have also been used in order to confirm the synthesis of ligands with different types of nanosystems. In studies with GL and GA, nuclear magnetic resonance (NMR) [80,81,82,83,84,85] and Fourier transform infrared spectroscopy (FTIR) [81,82,86,87,88] were commonly used.

The conjugation of GL/GA to the nanosystem surface occurs through covalent bonds between the ligand and the wall of the micelles or, in some cases, between the ligand, an intermediate molecule, and the surface material. The incorporation of these ligands may vary depending on the type of nanosystem. To conjugate GL to liposomes, for example, an intermediate molecule is commonly used to bind the ligand to the surface material of the micelles [67]. However, in polymeric nanoparticles, it is observed that in some cases, it is also possible to conjugate GL directly to the nanoparticles without using an intermediate [84]. In the case of other less conventional nanomaterials, such as nanofibers, GL can be directly conjugated to the drug to be vectored [89].

### 3.1. Synthesis and Characterization of GL/GA Nanosystems Conjugation

#### 3.1.1. Liposomes

Tsuji et al. worked with GL functionalized liposomes [72]. First of incorporating GL on liposomes surface, the synthesis of 30-stearyl GL compound (GLOSt) was carried out by ester formation using isoureas. The GLOSt structure was attributed to NMR. In ^1^H- and ^13^C-NMR spectral data of GLOSt, the presence of stearyl group was identified, assuming that the diglucuronide moiety of the GL molecule protrudes from the liposomal surface. The mean diameters of control liposomes and GLOSt conjugated liposomes were 54 ± 30 nm and 60 ± 30 nm, respectively [67,72].

In another study with GA, the synthesized compound was 3-succinyl-30-stearyl glycyrrhetinic acid (Suc-GAOSt), which had an amphiphilic characteristic that facilitated the incorporation, in high proportion, of Suc-GAOSt in the liposome lipid layer. The stearyl group was identified by ^1^H- and ^13^C-NMR analyses [80].

Chen et al. obtained GA surface-modified oxaliplatin liposomes using the film dispersion method. In this work, the liposomes with GA had a spherical shape with a mean diameter of 93.2 ± 3.1 nm and a zeta potential of −21.3 ± 2.9 mV [90]. Additionally, GAL-GA-LPs (GA liposomes modified with galactosylated derivative ligand) were produced with the film dispersion method, and the results indicated that the particle size decreased when the GA/blank liposomes proportion was optimized [73].

In another study, glycyrrhetinic acid was used to obtain the 3-galactosidase-30-stearyl deoxyglycyrrhetinic acid liposomes (DGA-O-GAL-LPs) via film dispersion method for the encapsulation of cantharidin (CTD) in liver-targeting liposomes as a potential treatment of hepatocellular carcinoma treatment [91]. ^13^C-NMR spectrum showed that the values of the chemical shifts in the C3 atom were shifted to a lower magnetic field (δ from 79.03 to 90.92), indicating that the esterification process between the deoxyglycyrrhetinic acid and acetobromo-d-galactose proceeded, while the peaks at δ 177.55, 170.53, 170.38, and 169.48 disappeared, proving that the deacetylation was obtained.

Sun et al. developed GA-modified liposomes (GA-LPs), where GA was first covalently bonded to the distal end of 1,2-distearoyl-sn-glycero-3-phosphoethanolamine-N-[amino(polyethylene glycol)] (DSPE-PEG) by amide and, second, loaded onto the liposome surface [92]. GA-LPs were then labeled with fluorescein isothiocyanate (FITC) or coumarin-6 (Cou6) to in vitro study the cellular uptake mechanism and clearance kinetics in the human liver cancer cell line (HepG2), proving the affinity of GA with the hepatocellular carcinoma cells.

#### 3.1.2. Polymeric Nanoparticles

Chitosan/poly(ethylene glycol)-glycyrrhetinic acid (CS/PEG-GA) nanoparticles were prepared by the ionic gelation method [78]. PEG-GA moiety influenced the particle size and the zeta potential of the nanoparticles, as evidenced by DLS and TEM analyses. As the PEG-GA concentration increased, the size of the nanoparticles increased, and the zeta potential decreased. However, a suitable concentration of PEG-GA was selected to maintain the CS/PEG-GA nanoparticles size in the 200 nm range to ensure the drug targeting the liver. 

The use of another polysaccharide, such as alginate (ALG) or hyaluronic acid (HA), instead of chitosan also generated similar results, where the particle size increased, and the zeta potential decreased as the amount of GA added to the nanoparticles increased [76,93].

**Figure 4:**
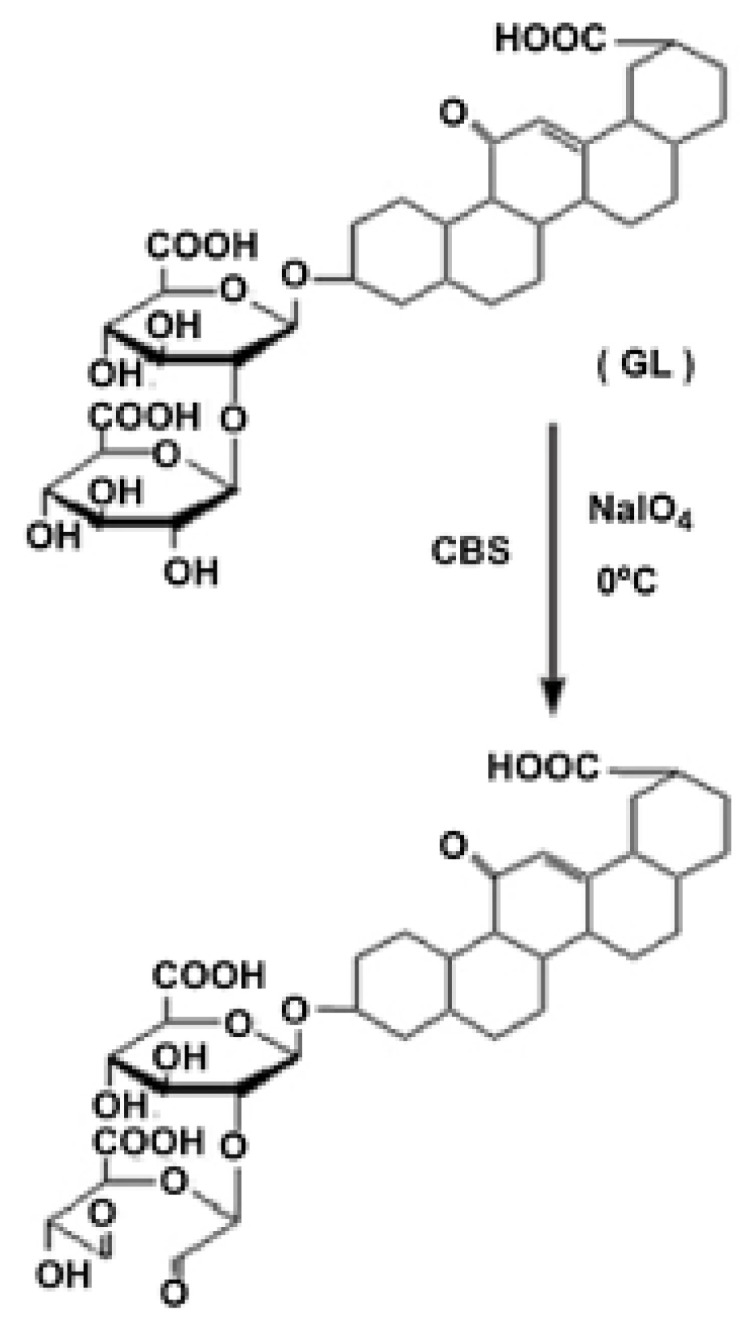
Oxidation reaction in carbonate buffered solution (pH 9.5) and cold sodium periodate solution (50 mM) to obtain the oxidized GL. Adapted with permission from [83], Elsevier, 2012.

The development of nanoparticles containing polysaccharides (ALG, CS, HA, etc.) has been the first choice for several researchers when GL had to be added on the surface of nanosystems for hepatocellular targeted drug delivery [75,76,77,93,94,95,96,97]. As also reported by Chen [89], the GL ligand has to be oxidized by means of a reaction with sodium periodate (NaIO_4_), allowing the formation of aldehyde groups (Figure 4). ^1^H-NMR analysis showed the appearance of two peaks at 8.19 and 8.5 ppm, corresponding to aldehyde protons [81,82,83,84]. Then, the aldehyde group interacts with the residual amino group on the surface of chitosan nanoparticles.

In the work performed by Lin and collaborators, the FT-IR analysis of glycyrrhizic acid surface-modified chitosan nanoparticles (GL-CS-NPs) evidenced the appearance of a new strong band at 1579 cm^−1^ and a significant decrease in the intensity of amine group absorption [81]. The new band was attributed to the formation of the C=N bond of the Schiff’s base due to the interaction between oxidized GL and CS-NPs. Additionally, the disappearance of the absorption peak of carboxyl group stretching vibration at 1731 cm^−1^, the appearance of the band at 1408 cm^−1^ (attributed to the COO^−^ symmetric stretching vibration), and the presence of absorption peak at 1631 cm^−1^ (assigned to the conjugated double bond vibrations in oxidized GL) were observed.

O-carboxymethyl chitosan nanoparticles (CMCS-NPs) were modified by GL [83]. The binding of oxidized-GL to CMCS-NPs was observed with FTIR analysis, being the peak representing symmetric stretching vibration of carboxyl groups shifted in oxidized-GL from 1351 to 1343 cm^−1^. Another way of confirming the interaction between the oxidized-GL and the amino groups present on the surface of chitosan nanoparticles was the use of ^1^H-NMR spectroscopy. Comparing the results from the analysis of chitosan nanoparticles and chitosan nanoparticles with GL surface-modified ones, it was possible to see the appearance of new peaks between 0.8 and 1.2 ppm due to the protons CH_3_, CH_2_, and CH of the steroid portion of GL [82].

The conjugation and insertion of the oxidized GL on the surface of nanoparticles were also observed in serum albumin (bovine or human) nanoparticles, having on their surface reactive amine groups that form the Schiff base through their conjugation with the aldehyde group of oxidized GL [98,99]. The confirmation of the conjugation was performed by FTIR analysis, being easily verified in the spectrum of glycyrrhizic acid-conjugated bovine serum albumin nanoparticles at 1053 cm^−1^ [99], while the characteristic peak of GL and glycyrrhizic acid-conjugated human serum albumin nanoparticles was observed at 1061 cm^−1^ [98].

Additionally, in the case of glycyrrhetinic acid-modified chitosan (GA-CS), the conjugation of GA with polysaccharides usually occurs via an ionic bond between the amine of CS and the carboxylic acid group of GA, as described by Cheng and collaborators [86]. FTIR spectrum revealed the disappearance of the GA carboxyl group absorption band (1706 cm^−1^); this was explained by the formation of the amide bond between the GA carboxyl group and the CS amine.

Another way is the conjugation of GA with CS by a click reaction, by the activation of GA through the formation of the O-acylisourea ester and its stabilization with the addition of N-hydroxysuccinimide, and then favoring the formation of amine in the presence of CS [87]. The conjugation was confirmed by ^1^H-NMR and FTIR analyses. In the NMR spectrum, the peak appearance at δ 1.8 ppm was attributed to the tertiary C9 proton of GA molecule, while the FTIR results showed the displacement of amides I and II bands to 1645 cm^−1^ and 1557 cm^−1^ with, in addition, an increase in the amide I peak intensity.

Tian et al. published two articles in 2011 and 2012. The first one had the objective of evaluating whether there was a difference in targeting effectiveness when the GA conjugation was performed on the C3-hydroxyl group or the C30-carboxyl group [100]. The synthesis of PEG-GA from the carboxyl group or the hydroxyl group of GA was performed. In the second article, a drug carrier based on glycyrrhetinic acid-modified sulfated chitosan was synthesized from the reaction of the amino group on the skeleton of sulfated chitosan with the carboxyl group in the C30 position of GA [75]. Regardless of which functional group (-hydroxyl group or -carboxyl group) of GA molecule was involved in the reaction, GA-modified nanoparticles had a similar trend to drug targeting to the liver.

ALG and HA nanoparticles functionalized with GA exhibit different formation modes due to the presence of functional groups that render their chemical structures negatively charged [93,96]. Thus, the use of an intermediate substance to allow ionic bonding between the polysaccharide and the vectorizing function component is required. In 2012, Zhang and collaborators published a study with doxorubicin-loaded glycyrrhetinic acid-modified alginate nanoparticles (DOX/GA-ALG-NPs), evaluating their bioavailability and antitumor activity in hepatic cells (H22) [93]. The conjugation of glycyrrhetinic acid with alginate was obtained by the covalent attachment of GA on the polysaccharide, which gave rise the formation of amide linkage between the carboxyl group of ALG and the amine group of GA. In a similar way, nanoparticles were prepared using histidine (HIS) to enable the conjugation between HA and GA [97]. A novel copolymer, named GHH, was synthesized using HA modified with GA and HIS. Then, the obtained GHH copolymer was used to prepare doxorubicin loaded nanoparticles (DOX/GHH) for liver-targeted drug delivery and pH-responsive drug release.

Wu et al. developed a hepatoma-targeting mixed micelles composed of hyaluronic acid–glycyrrhetinic acid conjugated and hyaluronic acid-L-histidine conjugate (HA–GA/HA–HIS) [101]. The structure of HA, GA, and HA–GA conjugate was confirmed via ^1^H-NMR. Chemical shifts corresponding to HA were observed at 2.0 and 3.3–4.7 ppm, and the characteristic peaks of GA protons were assigned at the 0.6–1.4 ppm range. The successful introduction of GA into HA was indicated by the presence of characteristic peaks at 0.6–1.4 ppm range. The degree of substitution was determined by comparing the average number of GA molecules attached per 100 HA molecules and was found to be within 3–20 by varying the ratio of GA to HA polymer.

In the study of Wang et al., liver targeting HA-GA succinate (HSG) nanoparticles were synthesized through the hydroxyl group modification of HA with succinate [96]. In order to retain the carboxyl groups of HA, succinic anhydride was selected as a bridge to couple HA with GA. A carboxyl group was introduced to the C3-hydroxyl group in GA using succinic anhydride; then, the carboxyl group of succinate-GA was covalently coupled with the hydroxyl group of HA. The characteristic peaks emerged in ^1^H-NMR and FT-IR analyses confirming the synthesis of HSG co-polymer and suggesting the GA-HA conjugation. ^1^H-NMR spectrum showed GA peaks at 0.8–1.7 ppm in the HSG spectrum, corresponding to the methyl and methylene groups. In the FITR spectrum of HSG, new peak appeared at 1729 cm^−1^ due to the formation of the ester carbonyl group, while the intensity of the band at 2930–2850 cm^−1^, attributed to the carbon-to-hydrogen stretching vibrations of methyl and methylene groups of GA moiety in HSG co-polymer, increased.

**Figure 5:**
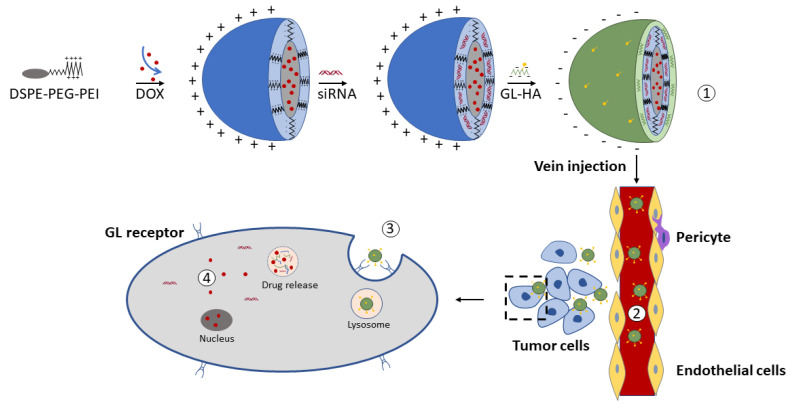
Schematic representation of (**1**) siRNA/DOX/GH-DPP-NPs preparation, (**2**) distribution of drugs vectorized to the liver by the blood cycle, (**3**) cell uptake, and (**4**) Bcl-2 siRNA and DOX release ph-triggered. Desaturated from [102], Frontiers, 2019.

Another possible way of promoting HA-GA conjugation was achieved through the use of a linker [102]. A liver-targeted DOX and Bcl-2 siRNA (short interfering RNA)-loaded nanoparticles, composed of 1,2-distearoyl-snglycero-3-phosphoethanolamine-polyethylene glycol-polyetherimide and glycyrrhetinic acid-modified hyaluronic acid (siRNA/DOX/GH-DPP-NPs), were prepared for drug combination therapy (Figure 5). The average particle size of siRNA/DOX/GH-DPP-NPs (185.4 ± 6.4 nm) was significantly bigger than that of siRNA/DOX/DPP NPs (157.2 ± 5.7 nm), while the zeta potential in siRNA/DOX/GH-DPP-NPs (−2.64 ± 1.73 mV) was decreased for approximately 15 mV compared to siRNA/DOX/DPP NPs. These results were due to the coverage of the nanoparticles with GA-HA conjugate, which generates particles of bigger size and lower zeta potential.

A study conducted by Cao et al. aimed to synthesize two hydrophobic targeting ligands modified for targeted DNA delivery to hepatocellular carcinoma [88]. Varied amounts of GA or GL were substituted to polyethyleneimine (PEI). Even the direct conjugation of PEI with GA or GL via the N-acylation pathway could improve its interaction with liver carcinoma and the gene transfection efficiency. The conjugation was confirmed by FTIR and ^1^H-NMR analyses: the FTIR band of -CO-NH- at 1649.5 cm^−1^ for GA or 1652.13 cm^−1^ for GL and the shifts at 5.5, 3.4–2.0 (-NHCH_2_CH_2_-), 2.0–0.43 ppm for GA, and 7.6 and 6.7 ppm (imidazole ring) for GL indicated the successful synthesis of PEI-GA and PEI-GL.

#### 3.1.3. Other Nanosystems

Some studies with unconventional nanosystems have also been performed. Liu et al. developed a polyamidoamine dendrimer (PAMAM) with GA based on the fact that the surface functional groups of graphene oxide (GO) have significant effects on the performance of GO-based gene delivery vector [74]. The amounts of free amino groups in the PAMAM with and without GA (GO-PAMAM-GA or GO-PAMAM, respectively) were determined by ninhydrin assays. In contrast to GO-PAMAM, the number of free amino groups in GO-PAMAM-GA decreased as the Ga content increased, demonstrating the functionalization of GO with PAMAM and GA molecules.

Chopdey et al. studied GL-conjugated polypropylene imine dendrimer (GL-PPI) and GL-conjugated multi-walled carbon nanotubes (GL-MWCNTs) as models of site-specific hepatic carriers for the delivery of DOX [94]. The conjugation was obtained by oxidation of GL to allow coupling of its carboxy group with the terminal NH_2_ group of PPI dendrimer or MWCNTs nanotubes.

Another example is represented by the preparation of a GA modified curcumin (CUR) supramolecular hydrogel, showing an increase in cellular uptake and greater inhibitory effect (smaller IC_50_ value) on HepG2 cells than the hydrogel without GA [82]. These results suggested the vectorizing effect of GA on liver cancer cells. An alternative type of micellar system for selective delivery of DOX to liver cancer cells is represented by GA-polymeric prodrug as a carrier for the encapsulation of DOX in self-assembled micelles (DOX/PEG-Fmoc-GA micelles) [103]. The coupling of GA to the unprotected amino groups of PEG-Fmoc-Lys, confirmed by NMR spectroscopy, led to the formation of the carrier that, through the thin film method, encapsulated DOX inside the self-assembled micelles, showing in vitro and in vivo antitumor effect.

The GA was also used for enhancing the cytotoxicity of allicin on hepatocellular cancer cell line HepG2 [104]. Allicin-loaded gelatin nanoparticles were conjugated to GA through amide bonds between the carboxyl group of GA and the primary amines of gelatin amino acids. The conjugation was confirmed by ^1^H-NMR spectroscopy, in which the disappearance of the OH carboxylic peak of GA (δ 11 ppm) was observed, confirming the link between the OH carboxylic of GA and the gelatin amino group.

### 3.2. Evaluation of Liver Drug Targeting

After the discovery of the affinity of GL for the liver [71] and the existence of specific GL and GA receptors [66,105] in the liver, the possibility of nanosystem vectors for liver-targeting was found. The efficiency of liver vectorization when GL or GA was added to the carrier was verified by in vitro and in vivo studies. The in vitro experiments involving nanocarriers and different types of cells were carried out to demonstrate that the presence of GL or GA increases the cellular uptake of the formulations.

In 1991 and 1994, the first studies demonstrating the vectorization of nanosystems (liposomes) to the liver by the introduction of GL molecules on the surface of the liposomes were published [66,72]. In these works, in vivo studies (male albino Wistar rats) showed that about 42% of the GLOSt-SUV (30-stearyl glycyrrhizic acid small unilamellar liposomes) injected dose was captured by the liver, presenting a 4-fold higher uptake than the control SUV (without GL); in vitro experiments (hepatocytes isolated from male albino Wistar rats) evidenced that the GLOSt-SUV uptake was 10-fold higher than the control SUV.

Nanoparticles with serum albumin (bovine or human) in the structure for transport and delivery of drugs also presented significant results in the vectorization to liver through the presence of GL on their surface. Mao et al. developed GL surface-modified calcein-loaded bovine serum albumin nanoparticles (CAL-BSA-GL-NPs) and tested their uptake in hepatocytes isolated from the normal liver of male Wistar rats [106]. It has been demonstrated that the modified nanoparticles with GL showed a 4-fold higher uptake than those unmodified with GL. In another study where bovine serum albumin was used, the human hepatocarcinoma cell line was incubated with GL nanoparticles labeled with isothiocyanate of fluorescein [99]. A higher fluorescence intensity of these formulations was observed compared to the same formulations without GL, thus demonstrating an increased uptake of GL-conjugated nanoparticles. Cell proliferation studies also showed high inhibitory rates of GL-conjugated nanoparticles over the unconjugated nanoparticles and the free drug (hydroxycamptothecin).

In a study in which human serum albumin was used, the lethality test performed by MTT assay on HepG2 showed that nanoparticles containing GL had a higher activity compared to the free drug (resveratrol (RES)) [85]. In vivo biodistribution tests in which the GL nanoparticles labeled with Cy5 NIR fluorophore were given to H22 tumor-bearing mice confirmed these results. Through the near-infrared fluorescence imaging technique, a significant drug accumulation in the liver of H22 tumor-bearing mice was observed after 72 h.

Flow cytometry and laser confocal microscopy techniques were used to detect the interaction of glycyrrhizic acid surface-modified chitosan nanoparticles (GL-CS-NPs), rhodamine B isothiocyanate labeled, with hepatocytes (parenchymal cells) and non-parenchymal liver cells [75]. The results showed that hepatocytes captured 4.9-fold more than non-parenchymal liver cells. A different result was observed for CS-NPs, which presented equal uptake to hepatocytes and non-parenchymal liver cells, reaffirming the selective uptake of GL-CS-NPs in hepatocytes. Another in vivo study demonstrated that the biodistribution of adriamycin-loaded GL-conjugated *N*-caproyl chitosan nanoparticles (ADR/GL-CCS-NPs) after injection in mice presented the drug accumulation mainly in the liver, spleen, and lungs [82]. ADR/GL-CCS-NPs obtained an uptake 1.6-fold higher than ADR/CCS-NPs in the liver and 2.1-fold higher in hepatocytes. In the case of GL-modified carboxymethyl chitosan nanoparticles loaded with paclitaxel (PTX/GL-CMC-NPs), the in vitro cytotoxicity study on human hepatocarcinoma cell line exhibited IC_50_ values of 2.7–3.2, 8.1, and 13.5 μg/mL for PTX/GL-CMC-NPs, PTX/CMC-NPs, and PTX, respectively, after 72 h of incubation [83]. The GL-conjugated nanoparticles were then more efficient on anti-proliferation; in addition, the cell uptake was 10-fold higher for PXT/GL-CMC-NPs compared to PXT/CMC-NPs. This trend was confirmed by the in vivo tests, in which the tumor inhibition rates were 87.5, 64.0, and 34.5% for PTX/GL-CMC-NPs, PTX/CMC-NPs, and PTX, respectively. In the work of El-Marakby, the effect of an injected dose in mice of valerate-conjugated chitosan, GL-decorated or undecorated, was evaluated [84]. Radiolabeled nanoparticles accumulated in the liver had a value of around 13% for GL-decorated nanoparticles and 4% for those non decorated, confirming the uptake increase in GL-decorated nanoparticles.

Another study, using GL for decoration, was performed with dendrimers and carbon nanotubes of multiple walls with the aim to obtain nanosystems for vectorization and delivery of drugs to the liver. In the hemolytic toxicity studies performed by Chopdey et al. with GL-PPI and GL-MWCNTs, both loaded with DOX, a significant reduction of DOX toxicity was obtained [98].

**Figure 6:**
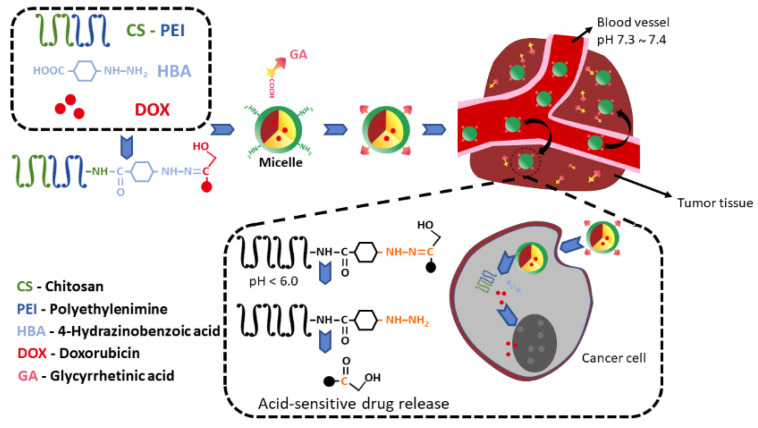
Schematic representation of acid-sensitive micelle with controlled drug release. The micelles decorated with GA would be easily accumulated in the tumor tissue by the EPR effect and internalized in the cancer cells. Adapted with permission from [77], Elsevier, 2018.

With regard to GA, in some cases, the amount of the compound detected inside the cells was two times higher when GA was present in the nanocarrier [75,77,90,101,107]. Experiments performed with cells that don’t present receptors for GA, such as MCF-7 and HUVEC (human umbilical vein endothelial), did not demonstrate any enhance in the number of nanocarriers into the cells when GA was present in the formulation, proving that the receptors of GA enhance the cellular intake (Figure 6) [77,101]. The introduction of GA to the micelles significantly increased the affinity of the formulations for HepG2 cells. Yang et al. also evidenced the synergistic effect of DOX and GA, loaded in polymeric micelle carrier, in inhibiting the proliferation of HepG2 cells [103].

The study with Hep3B hepatoma cell line and the human MDA-MB-231 breast cell line (used as a negative control) showed that the presence of GA into the micelles enhanced the uptake into Hep3B cells [108]. In the study carried out by Yang et al., DOX/PEG-Fmoc-GA micelles were more effective in inhibiting cell proliferation and inducing apoptosis due to the significant intracellular uptake of DOX by HepG2 cells when compared to a DOX solution [103]. Moreover, biodistribution studies with mice showed that the micelles were preferentially accumulated at the tumor site, demonstrating that the modification of the micelles with GA increased their affinity to liver cancer cells and enhanced their selective uptake by hepatocellular carcinoma cells.

Another example is represented by the synthesized lactobionic acid (LA)/lactoferrin (LF) or GA/LF conjugates used to coat sorafenib and quercitin (QRC) shell-oily core nanocapsules (NCs) to enhance their cellular internationalization via binding to asialoglycoprotein or GA receptors, respectively, on liver cancer cells [109]. The dual tumor-targeted NCs for co-drug delivery were in vitro and in vivo tested and compared with the free drugs. The in vitro cytotoxicity and cellular uptake studies on HepG2 liver cancer cells showed that LA/LF-NCs and GA/LF-NCs exhibited higher cytotoxic activity compared to the free drugs co-delivery. The in vivo antitumor efficacy of LA/LF-NCs and GA/LF-NCs in HCC-bearing mice reduced tumor proliferation due to their enhanced cellular uptake through LF-mediated endocytosis.

The ability to enhance the liver targeting was also noticed by Chen [73]. In vitro study with HepG2 cells showed that the amount of intracellular GA in GA-LPs and GAL-GA-LPs was greater than GA solution (GA-S). Zhu et al. developed a liposome of CTD, a potent drug against hepatocellular carcinoma, with a GA on the surface [79]. The pharmacokinetic and biodistribution experiments on Sprague–Dawley rats revealed that the presence of the GA in the liposome surface increased more than twice the concentration of the drug in the liver compared to the liposome without GA in the surface.

Another study performed by Tian and collaborators compared the uptake ratio of GA-liposomes in HepG2 cells and in human fetal hepatocyte (L-02) cells [110]. The liposomes of wogonin (WG), containing glycyrrhizic acid on the surface, presented a 2.5-fold higher cellular uptake in HepG2, displaying that the GA-modification of liposomes can distinguish hepatic carcinoma cells from normal hepatocytes. These results may indicate that more GA-specific binding sites are present on HepG2 cells than on L02 cells, resulting in increased endocytosis and WG uptake.

In the study of Zhou and collaborators, the liver-targeted GA-CTD liposome showed greater cytotoxicity and increased inhibition of HepG2 cell migration compared to the unmodified liposomes, which does not contain GA [91]. In addition, tissue distribution, efficiency relative targeting, uptake rate, and peak concentration ratio of CTD from the liver-targeted liposomes were higher in comparison to that of the unmodified liposome, concluding that the liposome modified with GA represents a promising nanocarrier for the drug targeted delivery to the liver.

Flow cytometry analysis was performed to compare endocytosis of nanoparticles containing DOX and recombinant human serum albumin (rHSA) in cells [111]. Targeted NPs containing GA showed higher binding to HepG2 cells than untargeted NPs. The mean fluorescence intensity of HepG2 cells treated with the DOX/GA-rHSA-NPs was higher than those incubated with nanoparticles not containing GA. Moreover, the receptor blocking with GA significantly reduced the fluorescence intensity of DOX/GA-rHSA-NPs. This result directly confirms that the cellular uptake of nanoparticles can be enhanced by attaching GA to their surface. Additionally, there was no difference in the fluorescence intensities between DOX/GA-rHSA-NPs and the DOX/rHSA-NPs for HeLa cells, i.e., GA receptor-negative cells. It was then concluded that the GA-targeted-NPs had a higher affinity to HepG2 than the untargeted NPs.

Mao and collaborators developed a calcein-loaded liposome surface modified with GA using the synthesized targeted molecule Suc-GAOSt and a calcein liposome without GA [80]. The cellular uptake of the Ga surface-modified liposome in the rat hepatocytes was 3.3 times higher when compared to the liposome without GA. Wang et al. studied nanocarriers with GA, PEI, and DNA to co-deliver drug and gene therapy, demonstrating that the enhanced uptake into HepG2 cells occurs through GA mediation [112]. This effect was also confirmed in a study in which the presence of GL or GA, conjugated with PEI, caused significant increases in the efficiency of gene transfection and superior selectivity for HepG2 cells [88].

To improve the drug bioavailability of QRC, Du et al. developed different drug-loaded self-aggregates of O-carboxymethyl chitosan-cholic acid conjugates with or without the presence of GA during the fabrication process of the nanocarrier, concluding that the presence of the GA enhanced the liver targeting [113].

Nanosystems decorated with GA also demonstrated that the presence of this ligand in the surface was able to target the nanocarriers to the liver, showing a great ability to permeate cancer cells and presenting a significant retention value at the site of action, thus confirming the great potential of GA as a vector of particles for the liver [90,93,108,113,114,115,116,117,118]. In addition to the studies in which the surface of chitosan nanoparticles was decorated with GA alone, other researchers decorated the surface of the nanoparticles with two ligands (GA and LA) able to increase the intracellular uptake of DOX [87]. Moreover, the serum biomarkers test (albumin, creatinine, urea, alpha-fetoprotein, alanine aminotransferase, aspartate aminotransferase, and alkaline phosphatase), together with the histopathological analysis of liver, kidney, and heart tissues, confirmed the safety of nanosystems with the two ligands compared to the conventional DOX.

In this context, several studies have been carried out with the intention of proving that the GA is capable of increasing the targeting of nanosystems to the liver compared to other organs of the body [86,108,111,119,120,121].

The amount of GA in the nanoparticles can be capable of generating an efficient cellular uptake, as demonstrated in a study conducted by Chu [122]. GA-modified curcumin-loaded nanostructured lipid carriers (CUR/GA-PEG-NLC) with different GA ratios (5%, 10%, and 15% *w/w*) were prepared to investigate the influence of GA on the cellular uptake and cytotoxicity against HepG2 cells. CUR/GA10%-PEG-NLC showed higher cellular uptake than the other two formulations. MMT assay evidenced higher cellular uptake and cytotoxicity of CUR/GA-PEG-NLC against HepG2 cells compared to the free drug.

In another study, a nanostructured lipid carrier was used to prepare liposomes (CUR-CA4P/GA-LPs) for liver-targeted co-delivery of curcumin and combretastatin A4 phosphate (CA4P), exploiting the apoptotic activity of curcumin with the antitumor angiogenic property of combretastatin [123]. The in vitro study on human hepatocellular carcinoma cell line (BEL-7402) showed the cellular uptake of CUR-CA4P/GA-LPs and an increased cytotoxicity activity of the two drugs when in combination in the liposomal structure. In in vivo antitumor efficacy of CUR-CA4P/GA-LPs in liver tumor, BALB7c male mice model was proven by higher tumor growth-inhibition, in addition to reducing systemic toxicity compared to free drugs. Moreover, the stronger tumor inhibition efficiency of CUR-CA4P/GA-LPs than that of CUR-CA4P/LPs would confirm the uptake of the GA-functionalized liposomes in the tumor cells through the GA-receptor mediated endocytosis.

In addition to increasing the absorption of drugs in healthy or cancerous liver cells, other studies showed the ability of GA to raise gene transfection [74,124]. Liu et al. evaluated the gene delivery capacity of the nanosystem constituted by GO with PAMAM and GA surface modification [74]. The obtained GO-PAMAM–GA hybrid facilitated the pEGFP-N1gene entering into the human hepatocarcinoma SMMC7721 cells; moreover, the efficiency of the hybrid to target the gene transfection into the SMMC-7721 cells rose with the increase in the GA amount.

Cytotoxicity tests on HepG2, Hep3B, L-02, HUVEC, and HELF (human fibroblast) cells were performed with antitumor drug- or gene-loaded GA micelles [77,95,114,115,124,125]. The micelles exhibited high lethality to HepG2 and Hep3B cells with enhanced inhibition on their proliferation via receptor-mediated endocytosis while presented low cytotoxicity to HUVEC, HELF, and L-02 cells. In confirmation of this, the cytotoxicity tests carried out with PTX/GA-g-HA-NPs (paclitaxel-loaded glycyrrhetinic acid-graft-hyaluronic acid nanoparticles) using HepG2 and B16F10 cells, a type of mouse melanoma cells, demonstrated a difference of drug concentration inside the two types of cell lines (HepG2 and B16F10) [114]. The higher cytotoxicity of PTX/GA-g-HA-NPs in HepG2 cells compared to B16F10 cells was due to the capacity of HepG2 cells to over-express both HA and GA receptors. Another example is represented by the cytotoxicity assessment of allicin-loaded gelatin nanoparticles GA-decorated in HepG2 cells that showed from 2- to 4-fold increase in allicin cytotoxicity compared to free drug, demonstrating that the conjugation of nanoparticles with GA was successful in vectoring the drug for hepatocellular carcinoma [104].

Histological evaluations revealed the capacity of DOX/GA-ALG-NPs to induce the death of cancer cells without affecting the surrounding healthy cells, showing a higher specificity of nanoparticles when compared to free DOX [93].

In another study, the targeting ability of GHH nanoparticles was evaluated with in vitro cellular uptake study by fluorescence microscopy, showing that the nanocarriers were internalized into the HepG2 cells [97]. The MTT test showed that the DOX/GHH nanoparticles exhibited a dose-dependent antitumor effect.

The viability of the cells A549 and HepG2 was also evaluated by MTT test after exposure with DOX nanoparticles and compared with free drug and GA [102,107]. The results suggested that the formulations present different cytotoxicity against HepG2 cells and A549 cells because of the different expressed levels of GA-receptor on these two different tumor cells.

The effect of GA-CS 5-flruorouracil nanoparticles (GA-CS/5-FU-NPs) on drug-resistant cells was investigated on the SMMC-7721 cell line, moderately resistant to 5-FU [86]. The results of this work demonstrated that GA-CS/5-FU-NPs significantly inhibited the proliferation of SMMC-7721 cells. Cytotoxicity in the GA-CS/5-FU-NPs group was significantly higher than in the 5-FU group. GA-CS nanoparticles enter cells by binding to GA receptors on the membrane, making the nanoparticles effective also against drug-resistant cells.

In vivo studies performed by Wu et al. using female BALB/c mice showed the efficient and rapid ability of the micelles with GA to target doxorubicin to the liver [101]. The in vivo pharmacokinetic studies with Sprague–Dawley rats using micelles of silybin with and without GA and silybin suspension showed that the micelles with GA achieved significant drug concentration in the liver [76]. Zhang et al. demonstrated that the micelles with GA exhibited significantly higher accumulation in the liver than in any other organs [108]. This indicates that GA modified micelles have higher retention time in vivo and exhibit liver-targeting property.

Du et al. investigated the behavior of QRC-loaded GA-decorated chitosan self-aggregates after IV administration in Wistar rats [113]. The presence of GA in the formulation provided the ability of the self-aggregates to target the rat liver. The mean residence time of the formulation containing GA was approximately eight times higher compared to the free drug, indicating a longer in vivo circulation time.

In vivo study on Kunming mice of GA-modified hyaluronic acid nanoparticles for intracellular delivery of DOX evidenced the preferential accumulation of the drug in the liver and hepatoma tissues [115]. Another in vivo study performed on Kunming mice with GA nanoparticles demonstrated two times higher drug accumulation in the liver than the Ga-free nanoparticles: the different results were attributed to the presence of GA receptors on hepatocytes [77]. Chen et al. performed an in vivo test with Kunming mice (male and female) demonstrating that after the intravenous administration of GA-S, GA-LP, and GAL-GA-LP, the concentration of GA in the liver was 2.5 times higher in the GAL-GA-LP mice group compared to GA-LP mice group, while the GA plasma concentration and tissue distribution from GA-S declined very fast [73]. The results evidenced the increase in liver targeting of the liposomes when GA was combined with GAL.

Tian et al. worked with H22 tumor-bearing mice analyzing the bio-distribution and antitumor therapeutic effects of siRNA/DOX/GH-DPP-NPs, concluding that the DOX and Bcl-2 siRNA combined therapy in the presence of GA improved antitumor efficacy [9]. GA-receptor-mediated internalization significantly increased the cellular uptake efficiency, and the Ga-nanoparticles could induce more cellular apoptosis.

Biodistribution studies of DOX nanoparticles in Balb/c were assessed by single-photon emission computed tomography [78]. The results demonstrated a 19 folds improvement in cell uptake of CS/PEG-GA nanoparticles. In a biodistribution study, Tian et al. identified that GA-WG-LPs could be accumulated rapidly in the tumor, liver, and spleen just 15 min after injection [110]. The uptake of these liposomes in the tumor was the highest among all of the excised tissues and was also much higher than the uptake of the free WG and nanoparticles without GA (WG-LPs). In the same work, the tumor inhibitory ratios of WG-LPs and GA-WG-LPs were 31.34% and 53.73%, respectively. The better in vivo antitumor results of the GA-WG-LPs occurred due to a larger cellular uptake via the GA-specific binding sites on the surface of hepatic cells, which improved liver targeting. GA-WG-LPs can actively and specifically target the liver, resulting in decreased tumor weight. An improvement in biodistribution, tumor accumulation, and therapeutic efficacy due to the increased receptor-mediated uptake of liposomes was observed in liver cells.

Wang et al. performed a study with Kunming mice and BALB/c nude mice. The results showed that the nanocarriers containing GA presented longer blood circulation and a significantly high accumulation in liver and liver tumors [96].

The mouse MDA-MB-231 xenograft model was used for the evaluation of in vivo anticancer activity of glycyrrhetinic acid-graft-hyaluronic acid nanoparticles considering the tumor growth and side effects on other tissues because of the enhanced cellular uptake of HGA nanoparticles in the cancer cells [114]. The study permitted to conclude that PTX/GA-g-HA-NPs showed high stability and good biocompatibility when compared to the free drug, inducing higher apoptosis of the cancer cells without unwanted side effects.

In summary, all the studies showed that the formulations including GL or GA can be successfully used for hepatocyte targeting, enhancing the activity of the drug in the nanosystems but also reducing the adverse effects of the anticancer drugs used in the treatment of hepatic cancer.

## 4. Conclusions

The search for new drugs has always been the major goal in the medical and pharmaceutical field due to the variate number of existing and newly discovered diseases. Plants have always been and are a great source of new molecules, which are studied and tested for new substances with pharmacological activities. However, some well-known molecules, such as GL and its metabolite GA, obtained from the *Glycyrrhiza glabra* plant, popularly known as licorice, are extensively investigated, seeking new pharmacological activities. These investigations sometimes lead to very significant discoveries, such as specific receptors that explain the greater safety and efficacy of some drugs in certain organs and the possibility of their use in combination with other drugs or delivery systems. As an example, the in vivo studies showed higher biodistribution of GL and GA in the liver, spleen, and lung, with the highest concentration in the liver. In vitro studies in the presence of specific receptors in the hepatocytes supported these results.

Based on the existence of specific receptors for GL and GA, which promote their internalization in liver cells, we presented in this review many recent published works both in vitro and in vivo. The decoration of the nanosystems surface (liposomes and various types of nanoparticles) with the objective to promote the vectorization of these nanosystems opens the delivery of antitumor drugs to the liver and liver tumor cells. Nanosystems decorated with GL or GA by different techniques, dependent on the constituents used in their manufacture, exhibited great success.

Through the use of oxidized glycyrrhizic acid, it was possible to decorate chitosan nanoparticles, serum albumin (human or bovine), dendrimer, and multi-walled carbon nanotubes by conjugation with the terminal -NH_2_ group. For liposomes, it was necessary to synthesize the compound GLOSt for GL and for GA, the compound Suc-GAOSt.

The vector capability of the GL- or GA-decorated nanosystems was demonstrated by the results of biodistribution or in vitro uptake compared to non-conjugated nanosystems. For example, in vivo studies for glycyrrhizic acid showed about 42% of intravenously injected dose retention of conjugated liposomes and 51% liver accumulation for glycyrrhetinic acid nanoparticles compared to 20% for unconjugated nanoparticles. For in vitro tests, cell uptakes were from 1.5 to 10 times higher than non-GL- or GA-conjugated nanosystems, with uptake percentage dependent on the nanosystem type. The studies presented also demonstrated that drug-free GL- or GA-conjugated nanosystems are safe and non-toxic in in vitro and in vivo studies. With regard to biological activity, in the GL- or GA-conjugated nanosystems, the drugs carried out the same therapeutic activity also at a low dosage and in some cases reduced known side effects for the free drug.

Taking into consideration the information described in this review, glycyrrhizic acid and glycyrrhetinic acid, due to receptors in the liver, have a great potential for the vectorization and delivery of nano-encapsulated antitumor drugs for the treatment of liver cancer, in addition to presenting low toxicity and high activity at low dosage.

## Data Availability

Not Applicable.

## Supplementary Materials

The following are available online at https://www.mdpi.com/article/10.3390/pharmaceutics13111792/s1, Table S1: Glycyrrhizic acid (GL) functionalized nanosystems for liver drug targeting, Table S2: Glycyrrhetinic acid (GA) functionalized nanosystems for liver drug targeting.

## Abbreviations

The following abbreviations are used in this manuscript.

ACE2: angiotensin-converting enzyme 2; ADR: adriamycin; ALG: alginate; ALI: acute lung injury; 11β-HSD2: 11β-hydroxysteroid dehydrogenase type 2 enzyme; BSA: bovine serum albumin; CA4P: combretastatin A4 phosphate; CAL: calcein; CAL-BSA-GL-NPs: glycyrrhizic acid surface-modified calcein-loaded bovine serum albumin nanoparticles; ADR/GL-CCS-NPs: adriamycin-loaded glycyrrhizic acid-conjugated *N*-caproyl chitosan nanoparticles; CMCS-NPs: O-carboxymethyl chitosan nanoparticles; CTD: cantharidin; CS: chitosan; CS/PEG-GA: chitosan/poly(ethylene glycol) glycyrrhetinic acid nanoparticles; Cou6: coumarin 6; CUR: curcumin; CUR/GA-ALB-NPs: curcumin-loaded glycyrrhetinic acid surface functionalized albumin nanoparticles; CUR/GA-PEG-NLC: curcumin-loaded glycyrrhetinic acid modified nanostructured lipid carrier; CUR-CA4P/GA-LPs: curcumin-combretastatin A4 phosphate glycyrrhetinic acid liposomes; CUR-CA4P/LPs: curcumin-combretastatin A4 phosphate liposomes; DGA-O-GAL-LPs: 3-galactosidase-30-stearyl deoxyglycyrrhetinic acid liposomes; DLS: dynamic light scattering; DOX: doxorubicin; DOX/GA-ALG-NPs: doxorubicin-loaded glycyrrhetinic acid-modified alginate nanoparticles; DOX/GHH: doxorubicin-loaded GHH nanoparticles; DOX/GA-rHSA-NPs: doxorubicin-loaded glycyrrhetinic acid modified recombinant human serum albumin nanoparticles; DOX/PEG-Fmoc-GA micelles: doxorubicin loaded glycyrrhetinic acid-conjugated polymeric prodrug micelles; DSPE-PEG: 1,2-distearoyl-sn-glycero-3-phosphoethanolamine-N-[amino(polyethylene glycol) - 2000]; EPR: enhanced permeability and retention; FER: ferulic acid; FITC: fluorescein isothiocyanate; 5-FU: 5-flruorouracil; FTIR: Fourier transformed infrared spectroscopy; GA: glycyrrhetinic acid; GA-CS: glycyrrhetinic acid-modified chitosan; GA-CS/CY-PCL: glycyrrhetinic acid-modified chitosan-cystamine-poly(e-caprolactone) copolymer; GA-CS/5-FU-NPs: 5-flruorouracil-loaded glycyrrhetinic acid-modified chitosan nanoparticles; GA-LPs: glycyrrhetinic acid-modified liposomes; GA-OX-LPs: oxaliplatin-loaded glycyrrhetinic acid liposomes; GA-S: glycyrrhetinic acid solution; GA-WG-LPs: wogonin-loaded glycyrrhetinic acid liposomes; GAL: galactosylated derivative ligand; GAL-GA-LPs: glycyrrhetinic acid liposomes modified with galactosylated derivative ligand; GHH: glycyrrhetinic acid-hyaluronic acid-histidine copolymer; GL: glycyrrhizic acid; GL-BSA-HCPT-NPs: 10-hydroxycamptothecin-loaded glycyrrhizic acid-conjugated bovine serum albumin nanoparticles; GL-CS-NPs: glycyrrhizic acid surface modified chitosan nanoparticles; GL-HSA-RES-NPs: resveratrol-loaded glycyrrhizic acid-conjugated human serum albumin nanoparticles; GL-MWCNTs: glycyrrhizin conjugated with multi-walled carbon nanotubes; GL-PPI: glycyrrhizic acid-conjugated polypropylene imine dendrimer; GA: glycyrrhetinic acid; GLOSt: 30-stearyl glycyrrhizin; GLOSt-SUV: 30-stearyl glycyrrhizin small unilamellar liposomes; GO: graphene oxide; GO-PAMAM-GA: polyamidoamine dendrimer and glycyrrhetinic acid functionalized on the dendrimer surface by graphene oxid; HA: hyaluronic acid; HA–GA/HA–HIS: glycyrrhetinic acid conjugated with hyaluronic/glycyrrhetinic acid acid-L-histidine; HCC: hepatocellular carcinoma; HCPT: 10-hydroxycamptothecin; HGA: glycyrrhetinic acid-graft-hyaluronic acid; HepG2: human liver cancer cell line; HIS: histidine; HMGB1: high mobility group box 1; HUVEC: human umbilical vein endothelial; IFN: interferon; IL: interleukin; INL: inulin; LA: lactobionic acid; LF: lactoferrin; LPs: liposomes; MR: mineralocorticoid receptors; MyD88: myeloid differentiation factor 88; NF-kB: nuclear factor kappa-light-chain-enhancer of activated B cells; NK: natural killer cells; NMR: nuclear magnetic resonance; OX: oxliplatin; PAMAM: polyamidoamine dendrimer; PEG: polyethylene glycol; PEG-GA: poly(ethylene glycol)-glycyrrhetinic acid; PEI: polyethyleneimine; PTX: paclitaxel; PTX/GA-g-HA-NPs: paclitaxel-loaded glycyrrhetinic acid-graft-hyaluronic acid nanoparticles; PTX/GL-CMC-NPs: paclitaxel-loaded glycyrrhizic acid modified carboxymethyl chitosan nanoparticles; QRC: quercetin; RAGE: receptor of advanced glycation end products; RES: resveratrol; rHSA: recombinant human serum albumin; ROS: reactive oxygen species; SARS-CoV-2: severe acute respiratory syndrome coronavirus 2; SIL: silybin; SCS: sulfated chitosan; siRNA: short interfering RNA; siRNA/DOX/GH-DPP-NPs: 2-distearoyl-snglycero-3-phosphoethanolamine-polyethylene glycol-polyetherimide and glycyrrhetinic acid-modified hyaluronic acid; STAT: signal transducer and activator of transcription; Suc-GAOSt: 3-Succinyl-30-stearyl glycyrrhetinic acid; TEM: transmission electron microscopy; TLR: toll-like receptor; TMPRSS2: cofactor transmembrane serine protease type 2; TNF-α: tumor necrosis factor alpha; VCS: valeric chitosan; WG: wogonin.

## References

[1] Bray F., Ferlay J., Soerjomataram I., Siegel R.L., Torre L.A., Jemal A. (2018). Global cancer statistics 2018: GLOBOCAN estimates of incidence and mortality worldwide for 36 cancers in 185 countries. CA Cancer J. Clin..

[2] Torre L.A., Siegel R.L., Ward E.M., Jemal A. (2016). Global cancer incidence and mortality rates and trends-an update. Cancer Epidemiol. Biomarkers Prev..

[3] Alsaleh M., Leftley Z., Barbera T.A., Sithithaworn P., Khuntikeo N., Loilome W., Yongvanit P., Cox I.J., Chamodol N., Syms R.R. (2019). Cholangiocarcinoma: A guide for the nonspecialist. Int. J. Gen. Med..

[4] Ghouri Y.A., Mian I., Rowe J.H. (2017). Review of hepatocellular carcinoma: Epidemiology, etiology, and carcinogenesis. J. Carcinog..

[5] Heimbach J.K., Kulik L.M., Finn R.S., Surlin C.B., Abecassis M.M., Roberts L.R., Zhu A.X., Murad M.H., Marrero J.A. (2018). AASLD guidelines for the treatment of hepatocellular carcinoma. Hepatology.

[6] Bosetti C., Turati F., La Vecchia C. (2014). Hepatocellular carcinoma epidemiology. Best Prac. Res. Clin. Gastroenterol..

[7] Valery P.C., Laversanne M., Clark P.J., Petrick J.L., Mcglynn K.A., Bray F. (2018). Projections of primary liver cancer to 2030 in 30 countries worldwide. Hepatology.

[8] Chen Z., Xie H., Hu M., Huang T., Hu Y., Sang N., Zhao Y. (2020). Recent progress in treatment of hepatocelular carcinoma. Am. J. Cancer Res..

[9] Zhang X., Ng H.L.H., Lu A., Lin C., Zhou L., Lin G., Zhang Y., Yang Z., Zhang H. (2016). Drug delivery system targeting advanced hepatocellular carcinoma: Current and future. Nanomed. Nanotechnol. Biol. Med..

[10] Cai Y., Xu Y., Chan H.F., Fang X., He C., Chen M. (2016). Glycyrrhetinic acid mediated drug delivery carriers for hepatocellular carcinoma therapy. Mol. Pharm..

[11] Su X., Wu L., Hu M., Dong W., Xu M., Zhang P. (2017). Glycyrrhizic acid: A promising carrier material for anticancer therapy. Biomed. Pharmacother..

[12] Xiong X., Gu L., Wang Y., Luo Y., Zang H., Lee J., Krams S., Zhu S., Zhao H. (2016). Glycyrrhizin protects against focal cerebral ischemia via inhibition of T cell activity and HMGB1-mediated mechanisms. J. Neuroinflamm..

[13] Li J., Xu H., Ke X., Tian J. (2012). The anti-tumor performance of docetaxel liposomes surface-modified with glycyrrhetinic acid. J. Drug Target..

[14] Li J.-Y., Cao H.-Y., Liu P., Cheng G.-H., Sun M.-Y. (2014). Glycyrrhizic acid in the treatment of liver diseases: Literature review. Biomed. Res. Int..

[15] EFSA (2015). Panel on Additives and Products or Substances used in Animal Feed (FEEDAP). Scientific opinion on the safety and efficacy of glycyrrhizic acid ammoniated (chemical group 30, miscellaneous substances) when used as a flavouring for all animal species. EFSA J..

[16] Van Rossum T.G., Vulto A.G., Hop W.C., Brouwer J.T., Niesters H.G., Schalm S.W. (1999). Intravenous glycyrrhizin for the treatment of chronic hepatitis C: A double-blind, randomized, placebo-controlled phase I/II trial. J. Gastroenterol. Hepatol..

[17] Bellussi L.M., Cocca S., Passali G.C., Passali D. (2017). HMGB1 in the Pathogenesis of Nasal Inflammatory Diseases and its Inhibition as New Therapeutic Approach: A Review from the Literature. Int. Arch. Otorhinolaryngol..

[18] Liu W., Huang S., Li Y., Li Y., Li D., Wu P., Wang Q., Zheng X., Zhang K. (2018). Glycyrrhizic acid from licorice down-regulates inflammatory responses via blocking MAPK and PI3K/Akt-dependent NF-κB signalling pathways in TPA-induced skin inflammation. MedChemComm.

[19] Qu L., Chen C., He W., Chen Y., Li Y., Wen Y., Zhou S., Jiang Y., Yang X., Zhang R. (2019). Glycyrrhizic acid ameliorates LPS-induced acute lung injury by regulating autophagy through the PI3K/AKT/mTOR pathway. Am. J. Transl. Res..

[20] Maione F., Minosi P., Di Giannuario A., Raucci F., Chini M.G., De Vita S., Bifulco G., Mascolo N., Pieretti S. (2019). Long-lasting anti-inflammatory and antinociceptive effects of acute ammonium glycyrrhizinate administration: Pharmacological, biochemical, and docking studies. Molecules.

[21] Bernela M., Ahuja M., Thakur R. (2016). Enhancement of anti-inflammatory activity of glycyrrhizic acid by encapsulation in chitosan-katira gum nanoparticles. Eur. J. Pharm. Biopharm..

[22] Han S., Sun L., He F., Che H. (2017). Anti-allergic activity of glycyrrhizic acid on IgE-mediated allergic reaction by regulation of allergy-related immune cells. Sci. Rep..

[23] Cinatl J., Morgenstern B., Bauer G., Chandra P., Rabenau H., Doerr H.W. (2003). Glycyrrhizin, an active component of liquorice roots, and replication of SARS-associated coronavirus. Lancet.

[24] Hoever G., Baltina L., Michaelis M., Kondratenko R., Baltina L., Tolstikov G.A., Doerr H.W., Cinatl J. (2005). Antiviral activity of glycyrrhizic acid derivatives against SARS-coronavirus. J. Med. Chem..

[25] Fu X., Wang Z., Li L., Dong S., Li Z., Jiang Z., Wang Y., Shui W. (2016). Novel chemical ligands to Ebola virus and Marburg virus nucleoproteins identified by combining affinity mass spectrometry and metabolomics approaches. Sci. Rep..

[26] Gupta P., Ukil A., Das P.K. (2017). Bioactive component of licorice as an antileishmanial agent. Biological Activities and Action Mechanisms of Licorice Ingredients.

[27] Chang H.-Y., Chen S.-Y., Wu C.-H., Yen G.-C. (2019). Glycyrrhizin attenuates the process of epithelial-to-mesenchymal transition by modulating HMGB1 initiated novel signaling pathway in prostate cancer cells. J. Agric. Food Chem..

[28] Tsai J.J., Pan P.J., Hsu F.T., Chung J.G., Chiang I.T. (2020). Glycyrrhizic acid modulates apoptosis through extrinsic/intrinsic pathways and inhibits protein kinase B-and extracellular signal-regulated kinase-mediated metastatic potential in hepatocellular carcinoma in vitro and in vivo. Am. J. Chin. Med..

[29] Khan R., Khan A.Q., Lateef A., Rehman M.U., Tahir M., Ali F., Hamiza O.O., Sultana S. (2013). Glycyrrhizic acid suppresses the development of precancerous lesions via regulating the hyperproliferation, inflammation, angiogenesis and apoptosis in the colon of Wistar rats. PLoS ONE.

[30] Zhang H., Huang Q., Zhai J., Zhao Y.N., Zhang L.P., Chen Y.Y., Zhang R.W., Li Q., Hu X.P. (2015). Structural basis for 18-β-glycyrrhetinic acid as a novel non-GSH analog glyoxalase I inhibitor. Acta Pharmacol. Sin..

[31] Chen J., Zhang Z., Song J., Liu Q., Wang C., Huang Z., Chu L., Liang H., Zhang B., Chen X. (2018). 18β-Glycyrrhetinic-acid-mediated unfolded protein response induces autophagy and apoptosis in hepatocellular carcinoma. Sci. Rep..

[32] Huo H.Z., Wang B., Liang Y.K., Bao Y.Y., Gu Y. (2011). Hepatoprotective and antioxidant effects of licorice extract against CCl4-induced oxidative damage in rats. Int. J. Mol. Sci..

[33] Kleiner D., Hegyi G., Urbanics R., Dezsi L., Robotka H., Feher E., Sardi E., Szebeni J., Blázovics A. (2016). Hepatoprotective liposomal glycyrrhizin in alcoholic liver injury. Eur. J. Int. Med..

[34] Huo X., Meng X., Zhang J., Zhao Y. (2020). Hepatoprotective effect of different combinations of 18α-and 18β-Glycyrrhizic acid against CCl4-induced liver injury in rats. Biomed. Pharmacother..

[35] Wu S.Y., Cui S.C., Wang L., Zhang Y.T., Yan X.X., Lu H.L., Xing G.Z., Ren J., Gong L.K. (2018). 18β-Glycyrrhetinic acid protects against alpha-naphthylisothiocyanate-induced cholestasis through activation of the Sirt1/FXR signaling pathway. Acta Pharmacol. Sin..

[36] Beskina O.A., Abramov A., Gabdulkhakova A.G., Miller A.V., Safronova V.G., Zamaraeva M.V. (2006). Possible mechanisms of antioxidant activity of glycyrrhizic acid. Biomed. Khim..

[37] Kao T.-C., Shyu M.-H., Yen G.-C. (2008). Neuroprotective effects of glycyrrhizic acid and 18β-glycyrrhetinic acid in PC12 cells via modulation of the PI3K/Akt pathway. J. Agric. Food Chem..

[38] Xu C., Liang C., Sun W., Chen J., Chen X. (2018). Glycyrrhizic acid ameliorates myocardial ischemic injury by the regulation of inflammation and oxidative state. Drug Des. Dev. Ther..

[39] Li M., Wen Z., Xue Y., Han X., Ma D., Ma Z., Wu Z., Guan S., Sun S., Chu L. (2020). Cardioprotective effects of glycyrrhizic acid involve inhibition of calcium influx via L-type calcium channels and myocardial contraction in rats. Naunyn Schmiedebergs Arch. Pharmacol..

[40] Lv X., Zhu Y., Deng Y., Zhang S., Zhang Q., Zhao B., Li G. (2020). Glycyrrhizin improved autophagy flux via HMGB1-dependent Akt/mTOR signaling pathway to prevent doxorubicin-induced cardiotoxicity. Toxicology.

[41] Ohnishi M., Katsuki H., Fukutomi C., Takahashi M., Motomura M., Fukunaga M., Matsuoka Y., Isohama Y., Izumi Y., Kume T. (2011). HMGB1 inhibitor glycyrrhizin attenuates intracerebral hemorrhage-induced injury in rats. Neuropharmacology.

[42] Ieong C., Sun H., Wang Q., Ma J. (2018). Glycyrrhizin suppresses the expressions of HMGB1 and ameliorates inflammative effect after acute subarachnoid hemorrhage in rat model. J. Clin. Neurosci..

[43] Paudel Y.N., Angelopoulou E., Semple B., Piperi C., Othman I., Shaikh M.F. (2020). Potential neuroprotective effect of the HMGB1 inhibitor glycyrrhizin in neurological disorders. ACS Chem. Neurosci..

[44] Bentz G.L., Lowrey A.J., Horne D.C., Nguyen V., Satterfield A.R., Ross T.D., Harrod A.E., Uchakina O.N., McKallip R.J. (2019). Using glycyrrhizic acid to target sumoylation processes during Epstein-Barr virus latency. PLoS ONE.

[45] Yu S., Liu M., Hu K. (2019). Natural products: Potential therapeutic agentes in multiple sclerosis. Int. Immunopharmacol..

[46] Selyutina O.Y., Polyakov N.E. (2019). Glycyrrhizic acid as a multifunctional drug carrier–from physicochemical properties to biomedical applications: A modern insight on the ancient drug. Int. J. Pharm..

[47] Paolino D., Lucania G., Mardente D., Alhaique F., Fresta M. (2005). Ethosomes for skin delivery of ammonium glycyrrhizinate: In vitro percutaneous permeation through human skin and in vivo anti-inflammatory activity on human volunteers. J. Control. Release.

[48] Bilia A.R., Bergonzi M.C., Guccione C., Manconi M., Fadda A., Sinico C. (2016). Vesicles and micelles: Two versatile vectors for the delivery of natural products. J. Drug Deliv. Sci. Technol..

[49] Shi J.R., Mao L.G., Jiang R.A., Qian Y., Tang H.F., Chen J.Q. (2010). Monoammonium glycyrrhizinate inhibited the inflammation of LPS-induced acute lung injury in mice. Int. Immunopharmacol..

[50] Andersson U., Tracey K.J. (2011). HMGB1 is a therapeutic target for sterile inflammation and infection. Annu. Rev. Immmunol..

[51] Qu L., Chen C., Chen Y.Y., Li Y., Tang F., Huang H., He W., Zhang R., Shen L. (2019). High-mobility group box 1 (HMGB1) and autophagy in acute lung injury (ALI): A review. Med. Sci. Monit..

[52] Li G., Liang X., Lotze M.T. (2013). HMGB1: The central cytokine for all lymphoid cells. Front. Immunol..

[53] Lee S., Kwak M.S., Kim S., Shin J. (2014). The role of high mobility group box 1 in innate immunity. Yonsei Med. J..

[54] Lee S.A., Lee S.H., Kim J.Y., Lee W.S. (2019). Effects of glycyrrhizin on lipopolysaccharide-induced acute lung injury in a mouse model. J. Thorac. Dis..

[55] Street M.E. (2020). HMGB1: A possible crucial therapeutic target for COVID-19. Horm. Res. Paediatr..

[56] Li C., Peng S., Liu X., Han C., Wang X., Jin T., Liu S., Wang W., Xie X., He X. (2017). Glycyrrhizin, a direct HMGB1 antagonist, ameliorates inflammatory infiltration in a model of autoimmune thyroiditis via inhibition of TLR2-HMGB1 signaling. Thyroid.

[57] Wang Y., Chen Q., Shi C., Jiao F., Gong Z. (2019). Mechanism of glycyrrhizin on ferroptosis during acute liver failure by inhibiting oxidative stress. Mol. Med. Rep..

[58] Shi X., Yu L., Zhang Y., Liu Z., Zhang H., Zhang Y., Liu P., Du P. (2020). Glycyrrhetinic acid alleviates hepatic inflammation injury in viral hepatitis disease via a HMGB1-TLR4 signaling pathway. Int. Immunopharmacol..

[59] Luo P., Liu D., Li J. (2020). Pharmacological perspective: Glycyrrhizin may be an efficacious therapeutic agent for COVID-19. Int. J. Antimicrob. Agents.

[60] Bailly C., Vergoten G. (2020). Glycyrrhizin: An alternative drug for the treatment of COVID-19 infection and the associated respiratory syndrome?. Pharmacol. Ther..

[61] Murck H. (2020). Symptomatic protective action of glycyrrhizin (licorice) in COVID-19 infection?. Front. Immunol..

[62] Cardone M., Yano M., Rosenberg A.S., Puig M. (2020). Lessons learned to date on COVID-19 hyperinflammatory syndrome: Considerations for interventions to mitigate SARS-CoV-2 viral infection and detrimental hyperinflammation. Front. Immunol..

[63] Tang D., Comish P., Kang R. (2020). The hallmarks of COVID-19 disease. PLoS Pathog..

[64] Falanga A., Tarallo R., Galdiero E., Cantisani M., Galdiero M., Galdiero S. (2013). Review of a viral peptide nanosystem for intracellular delivery. J. Nanophotonics.

[65] Demetzos C., Pippa N. (2014). Advanced drug delivery nanosystems (aDDnSs): A mini-review. Drug Deliv..

[66] Bayford R., Rademacher T., Roitt I., Wang S.X. (2017). Emerging applications of nanotechnology for diagnosis and therapy of disease: A review. Physiol. Meas..

[67] Osaka S., Tsuji H., Kiwada H. (1994). Uptake of liposomes surface-modified with glycyrrhizin by primary cultured rat hepatocytes. Biol. Pharm. Bull..

[68] Hu J.J., Xiao D., Zhang X.Z. (2016). Advances in peptide functionalization on mesoporous silica nanoparticles for controlled drug release. Small.

[69] Perez-Surio A.F., Alcarena-Lopez M.A. (2018). Drug vectoring systems to target drug delivery using nanotechnologies. Curr. Nanomed..

[70] Sun H., Dong Y., Feijen J., Zhong Z. (2018). Peptide-decorated polymeric nanomedicines for precision cancer therapy. J. Control. Release.

[71] Ichikawa T., Ishida S., Sakiya Y., Sawada Y., Hanano M. (1986). Biliary excretion and enterohepatic cycling of glycyrrhizin in rats. J. Pharm. Sci..

[72] Tsuji H., Osaka S., Kiwada H. (1991). Targeting of liposomes surface-modified with glycyrrhizin to the liver. I. Preparation and biological disposition. Chem. Pharm. Bull..

[73] Chen J., Chen Y., Chen Y., Gao Y., Zheng P., Li C., Tong Y., Li Z., Luo W., Chen Z. (2017). Modifying glycyrrhetinic acid liposomes with liver-targeting ligand of galactosylated derivative: Preparation and evaluations. Oncotarget.

[74] Liu F., Yang D., Lui Y., Cao Q., Sun Y., Wang Q., Tang H. (2018). Improving dispersive property, biocompatibility and targeting gene transfection of graphene oxide by covalente attachment of polyamidoamine dendrimer and glycyrrhetinic acid. Colloids Surf. B Biointerfaces.

[75] Tian Q., Wang X.-H., Wang W., Zhang C.-N., Wang P., Yuan Z. (2012). Self-assembly and liver targeting of sulfated chitosan nanoparticles functionalized with glycyrrhetinic acid. Nanomedicine.

[76] Han X., Wang Z., Wang M., Li J., Xu Y., He R., Guan H., Yue Z., Gong M. (2016). Liver-targeting self-assembled hyaluronic acid-glycyrrhetinic acid micelles enhance hepato-protective effect of silybin after oral administration. Drug Deliv..

[77] Yan T., Cheng J., Liu Z., Cheng F., Wei X., Huang Y., He J. (2018). Acid-sensitive polymeric vector targeting to hepatocarcinoma cells via glycyrrhetinic acid receptor-mediated endocytosis. Mater. Sci. Eng. C Mater. Biol. Appl..

[78] Tian Q., Zhang C.N., Wang X.H., Wang W., Huang W., Cha R.T., Wang C.H., Yuan Z., Liu M., Wan H.Y. (2010). Glycyrrhetinic acid-modified chitosan/poly(ethylene glycol) nanoparticles for liver-targeted delivery. Biomaterials.

[79] Zhu K., Zhou L., Zou M., Ning S., Liu S., Zhou Y., Du K., Zhang X., Xia X. (2020). 18-GA-Suc modified liposome loading cantharidin for augmenting hepatic specificity: Preparation, characterization, anti-Tumor effects and liver-targeting efficiency. J. Pharm. Sci..

[80] Mao S.J., Bi Y.Q., Jin H., Wei D.P., He R., Hou S.X. (2007). Preparation, characterization and uptake by primary cultured rat hepatocytes of liposomes surface-modified with glycyrrhetinic acid. Pharmazie.

[81] Lin A., Liu Y., Huang Y., Sun J., Wu Z., Zhang X., Ping Q. (2008). Glycyrrhizin surface-modified chitosan nanoparticles for hepatocyte-targeted delivery. Int. J. Pharm..

[82] Lin A., Chen J., Liu Y., Deng S., Wu Z., Huang Y., Ping Q. (2009). Preparation and evaluation of N-caproyl chitosan nanoparticles surface modified with glycyrrhizin for hepatocyte targeting. Drug Dev. Ind. Pharm..

[83] Shi L., Tang C., Yin C. (2012). Glycyrrhizin-modified O-carboxymethyl chitosan nanoparticles as drug vehicles targeting hepatocellular carcinoma. Biomaterials.

[84] El-Marakby E.M., Hathout R.M., Taha I., Mansour S., Mortada N.D. (2017). A novel serum-stable liver targeted cytotoxic system using valerate-conjugated chitosan nanoparticles surface decorated with glycyrrhizin. Int. J. Pharm..

[85] Wu M., Lia B., Deng Y., Feng Z., Zhong C., Wu W., Huang Y., Wang L., Zu C., Zhao X. (2017). Resveratrol-loaded glycyrrhizic acid-conjugated human serum albumin nanoparticles wrapping resveratrol nanoparticles: Preparation, characterization, and targeting effect on liver tumors. J. Biomater. Appl..

[86] Cheng M., Gao X., Wang Y., Chen H., He B., Xu H., Li Y., Han J., Zhang Z. (2013). Synthesis of glycyrrhenitic acid-modified chitosan 5-fluorouracil nanoparticles and its inhibition of liver câncer characteristics in vitro and in vivo. Mar. Drugs.

[87] Hefnawy A., Khalil I.H., Arafa K., Emara M., El-Sherbiny I.M. (2020). Dual-ligand functionalized core-shell chitosan-based nanocarrier for hepatocellular carcinoma-targeted drug delivery. Int. J. Nanomed..

[88] Cao M., Gao Y., Zhan M., Qiu N., Piao Y., Zhou Z., Shen Y. (2019). Glycyrrhizin acid and glycyrrhetinic acid modified polyethyleneimine for targeted DNA delivery to hepatocellular carcinoma. Int. J. Mol. Sci..

[89] Chen G., Li J., Cai Y., Zhan J., Gao J., Song M., Shi Y., Yang Z. (2017). A glycyrrhetinic acid-modified curcumin supramolecular hydrogel for liver tumor targeting therapy. Sci. Rep..

[90] Chen J., Jiang H., Wu Y., Li Y., Gao Y. (2015). A novel glycyrrhetinic acid-modified oxaliplatin liposome for liver-targeting and in vitro/vivo evaluation. Drug Des. Dev. Ther..

[91] Zhou L., Zou M., Zhu K., Ning S., Xia X. (2019). Development of 11-DGA-3-O-Gal-modified cantharidin liposomes for treatment of hepatocellular carcinoma. Molecules.

[92] Sun Y., Lu J., Yan D., Shen L., Hu H., Chen D. (2017). Cellular uptake mechanism and clearance kinetics of fluorescence-labeled glycyrrhetinic acid and glycyrrhetinic acid–modified liposome in hepatocellular carcinoma cells. Environ. Toxicol. Pharmacol..

[93] Zhang C., Wang W., Liu T., Wu Y., Guo H., Wang P., Tian Q., Wang Y., Yuan Z. (2012). Doxorubicin-loaded glycyrrhetinic acid-modified alginate nanoparticles for liver tumor chemotherapy. Biomaterials.

[94] Chen H., Li M., Wan T., Zheng Q., Cheng M., Huang S., Wang Y. (2012). Design and synthesis of dual-ligand modified chitosan as a liver targeting vector. J. Mater. Sci. Mater. Med..

[95] Yan T., Li D., Li J., Cheng F., Cheng J., Huang Y., He J. (2016). Effective co-delivery of doxorubicin and curcumin using a glycyrrhetinic acid-modified chitosan-cystamine-poly(ε-caprolactone) copolymer micelle for combination cancer chemotherapy. Colloids Surf. B Biointerfaces.

[96] Wang X., Gu X., Wang H., Sun Y., Wu H., Mao S. (2017). Synthesis, characterization and liver targeting evaluation of self-assembled hyaluronic acid nanoparticles functionalized with glycyrrhetinic acid. Eur. J. Pharm. Sci..

[97] Tian G., Sun X., Bai J., Dong J., Zhang B., Gao Z., Wu J. (2019). Doxorubicin-loaded dual-functional hyaluronic acid nanoparticles: Preparation, characterization and antitumor effcacy in vitro and in vivo. Mol. Med. Rep..

[98] Chopdey P.K., Tekade R.K., Mehra N.K., Mody N., Jain N.K. (2015). Glycyrrhizin conjugated dendrimer and multi-walled carbon nanotubes for liver specific delivery of doxorubicin. J. Nanosci. Nanotechnol..

[99] Zu Y., Meng L., Zhao X., Ge Y., Yu X., Zhang Y., Deng Y. (2013). Preparation of 10-hydroxycamptothecin-loaded glycyrrhizic acid-conjugated bovine sérum albumin nanoparticles for hepatocellular carcinoma-targeted drug delivery. Int. J. Nanomed..

[100] Tian Q., Wang X., Wang W., Zhang C., Yuan Z., Chen X. (2011). Understanding the role of the C3-hydroxyl group in glycyrrhetinic acid on liver targeting. J. Control. Release.

[101] Wu J.L., Tian G.X., Yu W.J., Jia G.T., Sun T.Y., Gao Z.Q. (2016). pH-responsive hyaluronic acid-based mixed micelles for the hepatoma-targeting delivery of doxorubicin. Int. J. Mol. Sci..

[102] Tian G., Pan R., Zhang B., Qu M., Lian B., Jiang H., Gao Z., Wu J. (2019). Liver-targeted combination therapy basing on glycyrrhizic acid-modified DSPE-PEG-PEI nanoparticles for co-delivery of doxorubicin and Bcl-2 siRNA. Front. Pharmacol..

[103] Yang T., Lan Y., Cao M., Ma X., Cao A., Sun Y., Yang J., Li L., Liu Y. (2019). Glycyrrhetinic acid-conjugated polymeric prodrug micelles co-delivered with doxorubicin as combination therapy treatment for liver cancer. Colloids Surf. B Biointerfaces.

[104] Ossama M., Hathout R.M., Attia D.A., Mortada N.D. (2019). Enhanced allicin cytotoxicity on HEPG-2 cells using glycyrrhetinic acid surface-decorated gelatin nanoparticles. ACS Omega.

[105] Negishi M., Irie A., Nagata N., Ichikawa A. (1991). Specific binding of glycyrrhetinic acid to the rat liver membrane. Biochim. Biophys. Acta.

[106] Mao S.J., Hou S.X., He R., Zhang L.K., Wei D.P., Bi Y.Q., Jin H. (2005). Uptake of albumin nanoparticle surface modified with glycyrrhizin by primary cultured rat hepatocytes. World J. Gastroenterol..

[107] Wang Q.S., Gao L.N., Zhu X.N., Zhang Y., Zhang C.N., Xu D., Cui Y.L. (2019). Co-delivery of glycyrrhizin and doxorubicin by alginate nanogel particles attenuates the activation of macrophage and enhances the therapeutic efficacy for hepatocellular carcinoma. Theranostics.

[108] Zhang J., Zhang M., Ji J., Fang X., Pan X., Wang Y., Wu C., Chen M. (2015). Glycyrrhetinic acid-mediated polymeric drug delivery targeting the acidic microenvironment of hepatocellular carcinoma. Pharm. Res..

[109] Abdelmoneem M.A., Elnaggar M.A., Hammady R.S., Kamel S.M., Helmy M.W., Abdulkader M.A., Zaky A., Fang J.Y., Elkhodairy A., Elzoghby A.O. (2019). Dual-targeted lactoferrin shell-oily core nanocapsules for synergistic targeted/herbal therapy of hepatocellular carcinoma. ACS Appl. Mater. Interfaces.

[110] Tian J., Wang L., Wang L., Ke X. (2014). A wogonin-loaded glycyrrhetinic acid-modified liposome for hepatic targeting with anti-tumor effects. Drug Deliv..

[111] Qi W.W., Yu H.Y., Guo H., Lou J., Wang Z.M., Liu P., Sapin-Minet A., Maicent P., Hong X.C., Hu X.M. (2015). Doxorubicin-loaded glycyrrhetinic acid modified recombinant human sérum albumin nanoparticles for targeting liver tumor chemotherapy. Mol. Pharm..

[112] Wang X., Niu D., Hu C., Li P. (2015). Polyethyleneimine-based nanocarriers for gene delivery. Curr. Pharm. Des..

[113] Du H., Liu M., Yang X., Zhai G. (2015). The role of glycyrrhetinic acid modification on preparation and evaluation of quercetin-loaded chitosan-based self-aggregates. J. Colloid Interface Sci..

[114] Zhang L., Yao J., Wang T., Zhang Q. (2013). Glycyrrhetinic acid-graft-hyaluronic acid conjugated as a carrier for synergistic targeted delivery of antitumor drugs. Int. J. Pharm..

[115] Mezghrani O., Tang Y., Ke X., Chen Y., Hu D., Tu J., Zhao L., Bourkaib N. (2015). Hepatocellular carcinoma dually-targeted nanoparticles for reduction triggered intracelular delivery of doxorubicin. Int. J. Pharm..

[116] Wang W., Lei Y., Sui H., Zhang W., Zhu R., Feng J., Wang H. (2017). Fabrication and evaluation of nanoparticle-assembled BSA microparticles for enhanced liver delivery of glycyrrhetinic acid. Artif. Cells Nanomed. Biotechnol..

[117] Wang X., Gu X., Huimin W., Yang J., Mao S. (2018). Enhanced delivery of doxorubicin to the liver through self-assembled nanoparticles formed via conjugation of glycyrrhetinic acid to the hydroxyl group of hyaluronic acid. Carbohydr. Polym..

[118] Li J., Chen T., Deng F., Wan J., Tang Y., Yuan P., Zhang L. (2015). Synthesis, characterization, and in vitro evaluation of curcumin-loaded albumin nanoparticles surface-functionalized with glycyrrhetinic acid. Int. J. Nanomed..

[119] Guo H., Lai Q., Wang W., Wu Y., Zhang C., Liu Y., Yuan Z. (2013). Functional alginate nanoparticles for eficiente intracelular release of doxorubicin and hepatoma carcinoma cell targeting therapy. Int. J. Pharm..

[120] Wu F., Li X., Jiang B., Yan J., Zhang Z., Qin J., Yu W., Gao Z. (2018). Glycyrrhetinic acid functionalized nanoparticles for drug delivery to liver cancer. J. Biomed. Nanotechnol..

[121] Du H., Yang X., Pang X., Zhai G. (2014). The synthesis, self-assembling, and biocompatibility of a novel O-carboxymethyl chitosan cholate decorated with glycyrrhetinic acid. Carbohydr. Polym..

[122] Chu Y., Li D., Luo Y.F., He X.J., Jiang M.Y. (2014). Preparation and in vitro evaluation of glycyrrhetinic acid-modified curcumin-loaded nanostructured lipid carriers. Molecules.

[123] Jiang H., Li Z.P., Tian G.X., Pan R.Y., Xu C.M., Zhang B., Wu J.L. (2019). Liver-targeted liposomes for codelivery of curcumin and combretastatin A4 phosphate: Preparation, characterization, and antitumor effects. Int. J. Nanomed..

[124] He Z.Y., Zheng X., Wu X.H., Song X.R., He G., Wu W.F., Yu S., Mao S.J., Wei Y.Q. (2010). Development of glycyrrhetinic acid-modified steath cationic liposomes for gene delivery. Int. J. Pharm..

[125] Marinez-Edo G., Fornaguera C., Borros S., Sanchez-Garcia D. (2020). Glycyrrhetinic acid-functionalized mesoporous silica nanoparticles for the co-delivery of DOX/CPT-PEG for targeting HepG2 cells. Pharmaceutics.

