# Supplementary material for: Glycyrrhizic Acid and Its Hydrolyzed Metabolite 18β-Glycyrrhetinic Acid as Specific Ligands for Targeting Nanosystems in the Treatment of Liver Cancer
Source: Pharmaceutics. 2021 Oct 26;13(11):1792. doi: 10.3390/pharmaceutics13111792 (PMC8621092; doi:10.3390/pharmaceutics13111792)
Supplement: Supplementary file 1 [file pharmaceutics-13-01792-s001.zip › pharmaceutics-1276556-supplementary.pdf]

pharmaceutics

MDPI

Supplementary Materials: Glycyrrhizic Acid and Its Hydrolyzed Metabolite 18β-Glycyrrhetinic Acid as Specific Ligands for Targeting Nanosystems in the Treatment of Liver Cancer

Luciano A. Stecanella, Antonio P.R. Bitencourt, Gustavo Richter Vaz, Eride Quarta, José O.C. Silva Júnior and Alessandra Rossi

Table S1. Glycyrrhizic acid (GL) functionalized nano-systems for liver drug targeting.

Nano-Systems	Drug	Formulation/Materials	Target/Characteristics	In Vitro/In Vivo Study	Main Results	Ref.
Liposome (GLOSt-SUV)	INL	Hydrogenated egg phosphatidylcholine-cholesterol-GLOSt or dicetyl phosphate Model drug: INL	Targeting therapy of liver cancer Rat hepatocytes Mean diameter: 60 nm	In vitro cellular uptake: interaction between GLOSt-SUV and hepatocytes In vivo in rats: determination of radioactivity in tissues, blood and urine samples.	GLOSt-SUV showed about a 10-fold higher uptake than the control-SUV during 2 h of incubation: GLOSt-SUV bind to the hepatocyte surface and are internalized and degraded in the cell. The clearance of GLOSt-SUV from blood after IV injection in rats was more rapid than that of control-SUV. GLOSt-SUV showed 4-fold higher uptake in the liver than the control; the blood clearance and the liver accumulation were dependent on the GLOSt-SUV content in liposomal membrane.	[1,2]
Dendrimer (GL-PPI) and Multi-Walled Carbon Nanotubes (GL-MWCNTs)	DOX	Dendrimer: ethylenediamine, acrylonitrile, polypropylene imine and GL Nanotubes: carbon, GL	Targeting therapy of hepatocellular carcinoma HepG2 cells Nanometric size, as evidenced by SEM and TEM analyses	In vitro drug release study, hemolytic and cytotoxicity assays	High DOX loading observed in DOX/GL-PPI and DOX/GL-MWCNTs (43 and 87 %, respectively). GL attachment reduced the hemolytic toxicity of DOX (12 and 7 % for DOX/GL-PPI and DOX/GL-MWCNTs, respectively). The IC_{50} of DOX was reduced from 4.2 μM to 2.0 μM and 2.7 μM, from DOX/GL-PPI and DOX/GL-MWCNTs, respectively. GL conjugated nanosystems were significantly dragging higher cancer cell number in early apoptosis as well as in early apoptotic phase.	[3]
GL surface modified albumin nanoparticles (GL-BSA-NPs)	HCPT	HCPT encapsulated in GL-surface modified bovine serum albumin nanoparticles	Targeting therapy of liver cancer Human hepatoma cells Z-Average size:158 nm Zeta Potential: −22.5 mV Drug encapsulation efficiency: 94 % Drug loading efficiency: 11 %	In vitro cellular uptake and cell proliferation assay Hemolysis test with rabbit blood	HCPT was wrapped in GL-BSA. In vitro drug release study showed slowly and continuously release. Cells incubated with HCPT/GL-BSA-NPs and labeled with fluorescein isothiocyanate showed stronger fluorescence intensity than samples without GL conjugation. The inhibitory rate of HCPT/GL-BSA-NPs developed as drug concentration was raised.	[4]
GL surface modified albumin nanoparticles (GL-BSA-NPs)	CAL	CAL encapsulated in GL-surface modified bovine serum albumin nanoparticles	Targeting therapy of liver cancer Rat hepatocytes Z-Average size: 79 nm Drug amount loaded in GL-BSA-CAL-NPs: 5.93 μg/mg	In vitro cellular uptake	The NPs cellular uptake in the hepatocytes reached its maximum at 2 h after incubation. The uptake amount of Cal/GL-BSA-NPs by rat hepatocytes was 4.43-fold higher than that of Cal/BSA-NPs due to the presence of specific GL binding site in the hepatocytes.	[5]
GL surface modified chitosan nanoparticles (GL-CS-NPs)	ADR	GL surface modified chitosan nanoparticles Model drug: ADR	Hepatocytes target Rat hepatic cells Mean diameter: 147 nm Zeta Potential: 9.3 mV Drug Association Efficiency: 91 %	In vitro drug release and cellular uptake	NPs showed in vitro low extent of release (28% over 72 h). GL-CS-NPs were preferentially accumulated in hepatocytes and the cellular uptake amount was 4.9 times than that in hepatic non parenchymal cells, and the uptake process was dependent on incubation time and dose of nanoparticles, indicating that the internalization of these nanoparticles into hepatocytes was mediated by a ligand–receptor interaction.	[6]

GL-conjugated *N*-caproyl chitosan nanoparticles (GL-CCS-NPs)	ADR	GL-conjugated *N*-caproyl chitosan nanoparticles<br>Model drug: ADR	Hepatocytes target<br>Hepatic cells<br>Mean diameter: 127 nm<br>Zeta Potential: 9.3 mV<br>Drug Association Efficiency: 87 %	In vitro: drug release in human plasma<br>In vivo: drug tissue distribution in kunming strain mice, hepatic cellular uptake	GL surface-modified NPs made ADR to dissolve and diffuse slowly to the plasma and led to low extent of release.<br>NPs formulations were accumulated in the liver and spleen. GL-CCS-NPs reached the highest level of targeting in the liver, nearly 1.6 times higher than that of non-GL-modified CCS-NPs. GL-CCS-NPs were preferentially distributed in hepatocytes by a ligand–receptor interaction.	[7]
Valeric chitosan nanoparticles (GL-VCS-NPs)	FER	Hydrophobically modified chitosan with valeric moiety<br>GL surface modified valeric chitosan nanoparticles<br>Model drug: FER	Active liver targeting<br>HepG2 cells<br>Z-Average size: 84 nm<br>Zeta Potential: 10.9 mV	In vitro cytotoxicity assay<br>In vivo biodistribution in albino mice	The GL surface decorated NPs showed highest cytotoxicity due to the presence of GL that may induce GL receptor mediated internalization.<br>The increased liver uptake of GL modified nanoparticles confirmed the recognition of nanoparticles by glycyrrhizin receptors on hepatocytes.	[8]
GL-conjugated human serum albumin nanoparticles (GL-HSA-NPs)	RES	GL coupled to human serum albumin<br>Resveratrol encapsulated in GL-conjugated HSA	Targeting therapy of liver cancer<br>HepG2 cells<br>Z-Average size: 108 nm<br>Drug encapsulation efficiency: 84 %<br>Drug loading efficiency: 11.5 %	In vitro: drug release study, lethality and targeting ability in HepG2 cells.<br>In vivo: biodistribution in H22 tumor-bearing mice	NPs slowly and continuously released the drug. The inhibitory rate of RES/GL-HAS-NPs was 62.5 mg/mL. The target ability of the NPs for HepG2 cells increased as NPs concentration raised.<br>The in vivo distribution study of labelled RES/GL-HSA-NPs exhibited significant drug accumulation in the liver of tumor-bearing mice.	[9]

Table S2. Glycyrrhetinic acid (GA) functionalized nano-systems for liver drug targeting.

Nano-Systems	Drug	Formulation/Materials	Target/CHARACTERISTICS	In Vitro/In Vivo Study	Main Results	Ref.
Liposomes Suc-GAOSt-LPs	CAL	3-Succinyl-30 stearyl GA liposome	Targeting therapy of liver cancer HepG2 cells Mean diameter: 68 nm	In vitro cellular uptake	Uptake of CAL/ Suc-GAOSt-LPs was 3.3-fold higher than that of CAL/LPs.	[10]
Nanostructured Lipid Carrier GA-PEG-NLC	CUR	GA-Modified CUR-Loaded Nanostructured Lipid Carrier	Targeting therapy of liver cancer HepG2 cells Particle size: 123 – 133 nm Zeta Potential: 14 – 16 mV Encapsulation efficiency: 90 –95%	In vitro cellular uptake by HPLC and cytotoxicity by MTT assay	CUR/GA-PEG-NLC have significantly high cellular uptake and cytotoxicity against HepG2 cells.	[11,12]
Lipid Nanoparticles GA-ALB-NPs	CUR	CUR loaded albumin nanoparticles surface-functionalized with GA	Targeting therapy of hepatocellular carcinoma HepG2 cells Z-Average size: 259 nm Encapsulation efficiency: 89%	In vitro cellular uptake by HPLC and cytotoxicity by MTT assay Apoptosis and cell cycle by FCM	CUR/GA-ALB-NPs are endocytosed into HepG2 cells, inducing cell cycle arrest in the G2/M phase. The observed number of apoptotic cells is consistent with the cytotoxicity seen over the observation period of 24 h.	[13]
Micelles GA-CS/CY-PCL	CUR DOX	GA-modified chitosan-cystamine-poly(e-caprolactone) copolymer micelle loaded with DOX and CUR	Targeting therapy of liver cancer HepG2 cells Z-Average size: 100 nm Zeta Potential: −30 mV Encapsulation efficiency of DOX and CUR: 20 and 9 %, respectively	In vitro cellular uptake by fluorescence assay and cytotoxicity by MTT assay	The cellular uptake is stronger when GA is present. The micelles exhibit enhanced inhibition on proliferation of the tested cancer cells. The improved efficiency is due to the synergy of DOX and CUR.	[14]
Nanoparticles GA-ALG-NPs	DOX	DOX loaded GA-modified alginate nanoparticles	Targeting therapy of liver cancer HepG2 cells Mean diameter: 274 nm Zeta Potential: 46 mV Loading Efficiency: 10%	In vitro: determination of cytotoxicity and cellular HepG2 cells compatibility by MTT test In vivo: tumor growth inhibitory activities in mice bearing H22 liver tumors in situ	Good liver targeting ability due to both passive targeting via the enhanced permeability and retention effects and the active targeting ability of GA. DOX/GA-ALG-NPs enhanced the antitumor activity of DOX against liver tumors in situ. By histological examination NPs induced cell death in the majority of liver tumor cells. Effectively inhibit the growth of liver tumors in situ and significantly reduce systemic side effects.	[15,16]
Nanoparticles GHH-HIS	DOX	Hyaluronic acid modified with GA and L-histidine and doxorubicin loaded nanoparticles	Targeting therapy of liver cancer HepG2 cells Z-Average size: 157–238 nm Zeta Potential: −10–14 mV Encapsulation efficiency: 87–91 %	In vitro cellular uptake and cytotoxicity by MTT assay In vivo antitumor activity and imaging study of H22 tumor-bearing mice by near-infrared fluorescence	In vitro cellular uptake indicates that the introduction of HIS to the HA backbone substantially increase the release rate of DOX from the lysosomes of HepG2 cells. In vivo antitumor activity analysis showed that the GHH nanoparticles exhibited higher antitumor efficacy than free DOX or DOX/GA-HA nanoparticles.	[16]
Lipid nanoparticles CS/PEG-GA	DOX	CS/PEG-GA nanoparticles	Targeting therapy of liver cancer HepG2 cells Z-Average size: 172 – 232 nm Zeta Potential: 12.7 – 36.5 mV Drug loading: 13%	In vitro cellular uptake and cytotoxicity by MTT assay In vivo in mice antitumor activity and biodistribution by single-photon emission computed tomography	About 74% of the human hepatic carcinoma cells (QGY-7703) shows uptake of the nanoparticles; The nanoparticles are greatly cytotoxic to QGY-7703 cells and inhibit tumor growth in H22 cell-bearing mice.	[17]
Lipid nanoparticles GA-SCS-NPs	DOX	GA-sulfated chitosan	Targeting therapy of liver cancer HepG2 cells Z-Average size: 183–177 nm Zeta Potential: −29.9 – −30.7 mV Encapsulation efficiency: 67–75 %	In vitro cellular uptake and cytotoxicity by MTT assay In vivo in mice antitumor activity and biodistribution	For the group injected with FITC-GA-SCTS5%, the uptake by the liver is 664 µg/g, the highest among all the tissue. The IC50 against HepG2 cells was 55 ng/mL; there was 2.2-fold improvement in uptake of the DOX micelles by HepG2 cells than that by Chang liver cells.	[9]
Nanoparticles GA-HSG-NPs	DOX	Self-assembled nanoparticles formed via conjugation of GA to the hydroxyl	Targeting therapy of liver cancer HepG2 cells Z-Average size: 180–280 nm Zeta Potential: −27–−35 mV;	In vitro cellular uptake by fluorescence assay and cytotoxicity by MTT assay In vivo in rats pharmacokinetic profile	The accumulation of DOX/GA-HSG-NPs in the liver was from 2.6 to 4.0-fold higher than that of DOX solution. In vivo imaging analysis further demonstrated nanoparticles not only have better liver targeting effect, but	[18]

		group of hyaluronic acid through succinic anhydride bridge	Encapsulation efficiency: 50–73 %	In vivo imaging study in tumor-bearing mice by near-infrared fluorescence.	also present superior tumor targeting efficiency than DOX solution. The DOX/GA-HSG-NPs and DOX solution have AUC of 50 and 2.1 mg/L·h and C_{max} of 19.6 and 1.8 mg/L, respectively.	
Liposomes GAL-GA-LPs		GA liposomes modified with a liver-targeting galactosylated derivative ligand	Targeting therapy of liver cancer HepG2 cells Mean diameter: 150 nm Zeta Potential: -35.5 mV Encapsulation Efficiency: >93%	In vitro cellular uptake by HPLC In vivo pharmacokinetic profile in mice	The amount of intracellular GAL-GA-LP is greater than GA-S suggesting that GA in liposomes increased HepG2 cellular uptake. The in vivo results show that the mean residence times and AUC (23.7 µg·h/L) of liposomes (GAL-GA-LP), is higher than the GA solution (8493 µg·h/L) (GA-S) in plasma.	[19]
Liposomes GA-LPs	OX	Liposomes surface modified with GA	Targeting therapy of Hepatocellular carcinoma HepG2 cells. Z-Average size: 93.2 nm Zeta Potential: −21.3 mV Encapsulation efficiency: >94%	In vivo in rats pharmacokinetic profile and histology studies.	The increased AUC from 240 to 996 mg·h/mL and C_{max} from 270 to 243 µg/mL of the GA-OX-LPs demonstrates an increased absorption than drug IV administration. The GA-modified liposomes deliver OX mainly to the liver. No severe signs, such as appearance of epithelial necrosis or sloughing of epithelial cells, are detected in the histology studies.	[20]
Nanoparticles HGA-NPs	PXT	GA-graft-hyaluronic acid loaded with paclitaxel.	Targeting therapy of liver cancer HepG2 cells Z-Average size: 321 nm Zeta Potential: −22.3–26.8 mV Encapsulation efficiency: 92%	In vitro cellular uptake by fluorescence assay and cytotoxicity by MTT assay In vivo imaging study in tumor-bearing mice by near-infrared fluorescence	The nanoparticles exhibit more significant cytotoxicity to HepG2 cells than B16F10 cells. The cellular uptake of nanoparticles is enhanced in HepG2 and B16F10 cells compared to a normal fibroblast cell (HELF cells). The liver and tumor targeting activity of nanoparticles is confirmed by in vivo imaging analysis. The fluorescence signals of nanoparticles in tumor and liver were 2.9 and 1.8-folds stronger than that of the control, respectively.	[21]
Micelles HA-ADH-DOCA HA-ADH-GA	SIL	Hyaluronic acid-deoxycholic acid (HA-ADH-DOCA) and hyaluronic acid-GA (HA-ADH-GA) conjugates	Targeting therapy of Acute liver injury Z-Average size: 122–128 nm Zeta Potential: −28–−29 mV Encapsulation efficiency: 92–93 %	In vivo in rats pharmacokinetic studies In vivo imaging study by near-infrared fluorescence	The AUC of 4.4 and 4.1 mg·h/mL and C_{max} of 0.88 µg/mL of the HA-ADH-DOCA and HA-ADH-GA micelles, respectively, demonstrates a significantly increased absorption than SIL suspension administration. In vivo imaging analysis confirms the liver targeting activity of micelles after oral administration. In comparison between the two micellar formulations, the fluorescence signals of HA-ADH-GA micelles in liver are stronger than that of HA-ADH-DOCA10 micelles.	[22]
Liposomes GA-LPs	WG	GA modified wogonin liposomes	Targeting therapy of Hepatocellular carcinoma HepG2 cells Z-Average size: 90 nm Zeta Potential: −16 mV Encapsulation efficiency: >92%.	In vitro cellular uptake and cytotoxicity by MTT assay In vivo in mice antitumor activity and biodistribution	GA-modified WG liposomes show the highest cellular uptake, at a rate of 1.6 times that of WG-LPs on HepG2 cells. HepG2 cell inhibitory efficacy of GA-WG-LPs IC_{50} is 1.5 times higher than that of WG-LPs. The tumour inhibitory ratios of GA-WG-LPs of 53.7% was significantly higher than WG-LPs.	[23]

References

1. Osaka, S.; Tsuji, H.; Kiwada, H. Uptake of liposomes surface-modified with glycyrrhizin by primary cultured rat hepatocytes. *Biol. Pharm. Bull.* **1994**, *17*, 940–943.
2. Tsuji, H.; Osaka, S.; Kiwada, H. Targeting of liposomes surface-modified with glycyrrhizin to the liver. I. Preparation and biological disposition. *Chem. Pharm. Bull.* **1991**, *39*, 1004–1008.
3. Chopdey, P.K.; Tekade, R.K.; Mehra, N.K.; Mody, N.; Jain, N.K. Glycyrrhizin conjugated dendrimer and multi-walled carbon nanotubes for liver specific delivery of doxorubicin. *J. Nanosci. Nanotechnol.* **2015**, *15*, 1088–1100.
4. Zu, Y.; Meng, L.; Zhao, X.; Ge, Y.; Yu, X.; Zhang, Y.; Deng, Y. Preparation of 10-hydroxycamptothecin-loaded glycyrrhizic acid-conjugated bovine sérum albumin nanoparticles for hepatocellular carcinoma-targeted drug delivery. *Int. J. Nanomed.* **2013**, *8*, 1207–1222.
5. Mao, S.J.; Hou, S.X.; He, R.; Zhang, L.K.; Wei, D.P.; Bi, Y.Q.; Jin, H. Uptake of albumin nanoparticle surface modified with glycyrrhizin by primary cultured rat hepatocytes. *World J. Gastroenterol.* **2005**, *28*, 3075–3079.

6. Lin, A.; Liu, Y.; Huang, Y.; Sun, J.; Wu, Z.; Zhang, X.; Ping, Q. Glycyrrhizin surface-modified chitosan nanoparticles for hepatocyte-targeted delivery. *Int. J. Pharm.* **2008**, *359*, 247–253.
7. Lin, A.; Chen, J.; Liu, Y.; Deng, S.; Wu, Z.; Huang, Y.; Ping, Q. Preparation and evaluation of N-caproyl chitosan nanoparticles surface modified with glycyrrhizin for hepatocyte targeting. *Drug Dev. Ind. Pharm.* **2009**, *35*, 1348–1355.
8. El-Marakby, E.M.; Hathout, R.M.; Taha, I.; Mansour, S.; Mortada, N.D. A novel serum-stable liver targeted cytotoxic system using valerate-conjugated chitosan nanoparticles surface decorated with glycyrrhizin. *Int. J. Pharm.* **2017**, *525*, 123–138.
9. Tian, Q.; Wang, X.-H.; Wang, W.; Zhang, C.-N.; Wang, P.; Yuan, Z. Self-assembly and liver targeting of sulfated chitosan nanoparticles functionalized with glycyrrhetinic acid. *Nanomedicine* **2012**, *8*, 870–879.
10. Mao, S.J.; Bi, Y.Q., Jin, H.; Wei, D.P.; He, R.; Hou, S.X. Preparation, characterization and uptake by primary cultured rat hepatocytes of liposomes surface-modified with glycyrrhetinic acid. *Pharmazie* **2007**, *62*, 614–619.
11. Chen, G.; Li, J.; Cai, Y.; Zhan, J.; Gao, J.; Song, M.; Shi, Y.; Yang, Z. A glycyrrhetinic acid-modified curcumin supramolecular hydrogel for liver tumor targeting therapy. *Sci. Rep.* **2017**, *7*, 44210.
12. Chu, Y.; Li, D.; Luo, Y.F.; He, X.J.; Jiang, M.Y. Preparation and in vitro evaluation of glycyrrhetinic acid-modified curcumin-loaded nanostructured lipid carriers. *Molecules* **2014**, *19*, 2445–2457.
13. Li, J.; Chen, T.; Deng, F.; Wan, J.; Tang, Y.; Yuan, P.; Zhang, L. Synthesis, characterization, and in vitro evaluation of curcumin-loaded albumin nanoparticles surface-functionalized with glycyrrhetinic acid. *Int. J. Nanomed.* **2015**, *10*, 5475–5487.
14. Yan, T.; Li, D.; Li, J.; Cheng, F.; Cheng, J.; Huang, Y.; He, J. Effective co-delivery of doxorubicin and curcumin using a glycyrrhetinic acid-modified chitosan-cystamine-poly(ε-caprolactone) copolymer micelle for combination cancer chemotherapy. *Colloids Surf. B Biointerfaces* **2016**, *145*, 526–538.
15. Zhang, C.; Wang, W.; Liu, T.; Wu, Y.; Guo, H.; Wang, P.; Tian, Q.; Wang, Y.; Yuan, Z. Doxorubicin-loaded glycyrrhetinic acid-modified alginate nanoparticles for liver tumor chemotherapy. *Biomaterials* **2012**, *33*, 2187–2196.
16. Tian, G.; Sun, X.; Bai, J.; Dong, J.; Zhang, B.; Gao, Z.; Wu, J. Doxorubicin-loaded dual-functional hyaluronic acid nanoparticles: Preparation, characterization and antitumor effcacy in vitro and in vivo. *Mol. Med. Rep.* **2019**, *19*, 133–142.
17. Tian, Q.; Zhang, C.N.; Wang, X.H.; Wang, W.; Huang, W.; Cha, R.T.; Wang, C.H.; Yuan, Z.; Liu, M.; Wan, H.Y.; et al. Glycyrrhetinic acid-modified chitosan/poly(ethylene glycol) nanoparticles for liver-targeted delivery. *Biomaterials* **2010**, *31*, 4748–4756.
18. Wang, X.; Gu, X.; Huimin, W.; Yang, J.; Mao, S. Enhanced delivery of doxorubicin to the liver through self-assembled nanoparticles formed via conjugation of glycyrrhetinic acid to the hydroxyl group of hyaluronic acid. *Carbohydr. Polym.* **2018**, *195*, 170–179.
19. Chen, J.; Chen, Yu.; Chen, Yi; Gao, Y.; Zheng, P.; Li, C.; Tong, Y.; Li, Z.; Luo, W.; Chen, Z. Modifying glycyrrhetinic acid liposomes with liver-targeting ligand of galactosylated derivative: Preparation and evaluations. *Oncotarget* **2017**, *8*, 102046–102066.
20. Chen, J.; Jiang, H.; Wu, Y.; Li, Y.; Gao, Y. A novel glycyrrhetinic acid-modified oxaliplatin liposome for liver-targeting and in vitro/vivo evaluation. *Drug Des. Dev. Ther.* **2015**, *9*, 2265–2275.
21. Zhang, L.; Yao, J.; Wang, T.; Zhang, Q. Glycyrrhetinic acid-graft-hyaluronic acid conjugated as a carrier for synergistic targeted delivery of antitumor drugs. *Int. J. Pharm.* **2013**, *30*, 654–664.
22. Han, X.; Wang, Z.; Wang, M.; Li, J.; Xu, Y.; He, R.; Guan, H.; Yue, Z.; Gong, M. Liver-targeting self-assembled hyaluronic acid-glycyrrhetinic acid micelles enhance hepato-protective effect of silybin after oral administration. *Drug Deliv.* **2016**, *23*, 1818–1829.
23. Tian, J.; Wang, L.; Wang, L.; Ke, X. A wogonin-loaded glycyrrhetinic acid-modified liposome for hepatic targeting with antitumor effects. *Drug Deliv.* **2014**, *21*, 553–559.
